# The impact of one-to-one peer support on mothers’ personal breastfeeding goals and emotional well-being: a scoping review

**DOI:** 10.1017/S1368980026102985

**Published:** 2026-07-07

**Authors:** Khayla Timothy, Elizabeth J. O’Sullivan, Áine O’Connor

**Affiliations:** 1 Sport & Health Sciences, Technological University of the Shannon: Midlands, Athlone Campus, Ireland; 2 School of Biological, Health and Sports Sciences, Technological University Dublin, Ireland

**Keywords:** Breastfeeding, Peer support, Mothers, Maternal emotional well-being, Scoping review

## Abstract

**Objective:**

To examine the impact of one-to-one peer support on mothers’ personal breastfeeding goals.

**Design:**

Scoping review guided by Arksey and O’Malley’s five-stage framework and reported in accordance with PRISMA Extension for Scoping Reviews guidelines. Qualitative data were analysed using descriptive content analysis. Quantitative data were analysed by identifying numerical trends and recurring patterns, and a concise overview of key descriptive findings was provided using frequency counts and proportions.

**Setting:**

Studies conducted across ten countries globally, identified through systematic searches of seven electronic databases and screening of reference lists.

**Participants:**

Thirty-eight studies were included: twenty quantitative, seven qualitative, six mixed methods and three secondary analyses (drawing on two relevant primary sources). Participants were mothers who received one-to-one breastfeeding peer support, predominantly in community or home-based settings.

**Results:**

One primary outcome was assessed: The impact of one-to-one peer support on mothers’ personal breastfeeding goals. Two secondary outcomes were identified. The first examined the effect of one-to-one peer support on breastfeeding outcomes based on traditional measures of breastfeeding success. Of the included studies, 50 % reported positive effects of one-to-one peer support on traditional measures of breastfeeding success, while 21 % found no statistically significant differences. An additional secondary outcome reported in 34 % of the included studies examined the impact of mother-centred breastfeeding peer support on maternal emotional well-being.

**Conclusions:**

One-to-one peer support enhances the mothers’ ability to achieve their personal breastfeeding goals and positively influences emotional well-being. These findings underscore the need to integrate structured one-to-one peer support into maternal health services in Ireland and globally.

Breastfeeding is the natural and biologically normal way of feeding infants and young children, ensuring optimum growth and development^(1)^. Breastfeeding offers a range of health benefits for mothers and infants. Infants who are breastfed have a reduced risk of developing asthma, obesity, type 1 diabetes and sudden infant death syndrome. Significant health benefits for mothers include a reduced risk of developing breast and ovarian cancers, type 2 diabetes, hypertension^(2)^ and cardiovascular disease^(3)^. Breastfeeding is an environmentally sustainable practice with little or no waste, has a low carbon footprint and helps conserve natural resources^(4)^.

National and international recommendations from both the Health Service Executive, WHO and UNICEF state that infants should be exclusively breastfed (EBF) for the first six months of life, with continued breastfeeding to 2 years and beyond, along with complementary foods^(5,6)^. However, despite these recommendations, Ireland has one of the lowest breastfeeding rates worldwide. In 2022, only 44 % of infants in Ireland were EBF upon discharge from maternity services^(7)^.

Women’s personal breastfeeding duration goals, whether those were met and their satisfaction with their own breastfeeding journey, has been minimally explored both in Ireland and elsewhere. A 2022 survey found that among over 5000 women who intended to breastfeed, just over 40 % planned to breastfeed for up to six months, 22 % aimed to breastfeed for up to one year, and 25 % did not have a specific breastfeeding goal^(8)^. The misalignment between women’s personal breastfeeding goals and the recommendations from public health bodies suggests that current metrics for determining breastfeeding success used by researchers and public health authorities may not always be appropriate. While it remains important to encourage higher prevalence of breastfeeding, it is arguably equally important to celebrate women’s individual achievements and support women in feeling positive about their breastfeeding journey.

Breastfeeding peer supporters have an important role to play in supporting mothers to work towards achieving their personal breastfeeding goals. Peer supporters are women with personal breastfeeding experience who offer practical support and emotional encouragement to other breastfeeding mothers^(9,10)^. By sharing lived experiences and providing guidance, they help new mothers navigate challenges and gain confidence in their breastfeeding journey^(9,10)^. Peer supporters may provide support through group settings or one-to-one interactions^(11)^. Evidence suggests that women value the approachability and non-judgmental, woman-centred approach of peer supporters, which helps to build maternal confidence, support emotional well-being and increase satisfaction with their breastfeeding journey^(12)^. Unlike healthcare professionals, who are often constrained by clinical targets and time pressures^(13)^, peer supporters can provide flexible, individualised support that validates women’s personal breastfeeding goals rather than relying solely on breastfeeding rates or duration to define success.

Previous quantitative research has explored the effectiveness of peer support (PS) in improving breastfeeding outcomes, based on traditional metrics of success. A systematic review from 2017 found that community-based breastfeeding PS not only increased the duration of EBF, particularly for infants aged three to six months in low- and middle-income countries, but also encouraged mothers to initiate breastfeeding earlier and decreased pre-lacteal feeding^(14)^. However, some trials did not find any significant benefit of PS on breastfeeding rates at time periods such as 10–14 d, 6 weeks or 6 months^(15)^ and initiation rates for breastfeeding did not differ between the intervention group (IG) (69·0 %) and control group (CG) (68·1 %) in another trial^(16)^.

In Ireland, previous research has primarily examined the effectiveness of breastfeeding support groups^(17,18)^ and support provided by healthcare professionals^(19,20)^. To date, however, no published studies to our knowledge have explored the specific impact of one-to-one PS in the Irish context. There is currently limited understanding of how one-to-one PS is experienced by mothers or of the barriers and facilitators that influence its delivery, acceptability and perceived value. Exploring this form of support is important, as one-to-one PS may function as an additional and more individualised model of breastfeeding support alongside existing group-based and professional services. This is particularly important given evidence that, while women are encouraged to breastfeed during pregnancy, many experience limited postnatal breastfeeding support in practice^(8)^. Addressing this gap will contribute to a more comprehensive understanding of how PS can best be integrated into breastfeeding support pathways in Ireland.

The rationale for this review is to explore the impact of one-to-one PS on mothers’ personal breastfeeding goals, building on previous work in this area^(21,22)^. By focusing specifically on mothers’ perspectives of receiving one-to-one breastfeeding PS from the outset, this review aims to provide a deeper understanding of the role peer supporters play in helping women work towards achieving their personal breastfeeding goals and how such support may influence satisfaction with their breastfeeding journey as opposed to focusing solely on targets imposed by public health bodies that define breastfeeding success in terms of breastfeeding rates or duration.

## Methods

A scoping review methodology was chosen to comprehensively examine the extent of existing literature on this topic, identify gaps in the literature and provide recommendations for future research^(23,24)^. Given the variability in how PS is delivered, experienced and evaluated across different contexts, a scoping review allows for the inclusion of a diverse range of study designs and a broad exploration of key concepts^(23,25)^.

The ‘PRISMA Extension for Scoping Reviews’ (PRISMA-ScR) was used to structure the reporting of this review, ensuring transparency, rigour and comprehensive documentation^(26)^. This tool provides a checklist of 20 essential reporting items and two optional items, aiding in systematically identifying, selecting and charting relevant literature while maintaining methodological clarity and consistency^(26)^.

In addition to PRISMA-ScR,^(26)^ this scoping review is guided by the framework of Arksey and O’Malley^(27)^. This approach includes five steps: defining the research question, identifying relevant studies, selecting studies, charting the data and analysing, summarising and reporting the findings^(27,28)^.

### Identifying the research question

The research question was developed using the search strategy tool ‘PEO’^(29)^. The PEO framework represents the population, exposure and outcomes of the research question, exhibited in Table 1. This review aimed to explore the research question: What impact does one-to-one peer breastfeeding support have on mothers’ personal breastfeeding goals?

**Table 1.** PEO framework Table 1 long description.

PEO	Definition	Example
**P**opulation	Who is my question focused on?	Mothers
**E**xposure	What is the issue I’m interested in?	Breastfeeding goals
**O**utcome	What, in relation to the issue, do I want to examine?	Peer support

### Identification of relevant studies

To obtain a greater variety of literature, a thorough search strategy was built based on the keywords generated from applying the PEO framework^(27,29)^. A comprehensive computer-assisted database search was conducted using seven electronic databases (CINAHL, Academic Search Complete, MEDLINE, Scopus, PsycINFO, EMBASE and Cochrane Library). Searches were performed using the Title OR Abstract and Keyword options, with each search string searched individually and subsequently all four strings combined. The same search terms were inputted in all databases and are presented in Table 2. Search techniques included the use of an asterisk (*) as a truncation symbol, placed at the end of a word root to prompt the database to retrieve all possible variations of the term^(30)^. Boolean operators were also applied with ‘OR’ used to broaden the results and ‘AND’ to narrow the results^(29)^. No limits (e.g. publication year or country) were applied to the search strategy, other than restricting studies to English-language publications.

**Table 2.** Search strategy Table 2 long description.

	Search terms
**S1 (String 1)**	**TI** (mother* OR maternal OR mom OR mum OR woman* OR women*) **OR AB** (mother* OR maternal OR mom OR mum OR woman* OR women*)
**S2 (String 2/Keyword S1)**	(‘Mother*’)
**S3 (String 3)**	**TI** (breastfeed* OR ‘breast-feed*’ OR ‘breast feed*’ OR breastfed OR ‘human-milk’ OR ‘human-milk feeding’ OR ‘breast milk’ OR ‘natural feeding’ OR ‘infant feeding’ OR ‘lactati*’) **OR AB** (breastfeed* OR ‘breast-feed*’ OR ‘breast feed*’ OR breastfed OR ‘human-milk’ OR ‘human-milk feeding’ OR ‘breast milk’ OR ‘natural feeding’ OR ‘infant feeding’ OR ‘lactati*’)
**S4 (String 4/Keyword S3)**	(‘Breast Feeding’)
**S5 (String 5)**	**TI** (goal* OR objective* OR target* OR aim* OR milestone* OR intention* OR aspiration* OR ambition* OR ‘desired outcome*’ OR purpose * OR ‘personal goal’ OR ‘personal ambition*’ OR ‘self-efficacy’) **OR AB** (goal* OR objective* OR target* OR aim* OR milestone* OR intention* OR aspiration* OR ambition* OR ‘desired outcome*’ OR purpose * OR ‘personal goal’ OR ‘personal ambition*’ OR ‘self-efficacy’)
**S6 (String 6/Keyword S5)**	(‘Goals’)
**S7 (String 7)**	**TI** (‘peer support*’ OR ‘peer-support*’ OR ‘peer group*’ OR ‘social support*’ OR ‘support group*’ OR ‘community support*’ OR ‘peer counsel*’ OR ‘peer mentor*’ OR ‘peer assist*’ OR ‘mother-to-mother support’ OR ‘one-to-one peer support’ OR ‘individual peer support’ OR ‘peer-led support’ OR ‘supportive interaction*’ OR ‘peer educator’ OR ‘peer listener’ OR ‘peer advocate’ OR ‘postpartum support’ OR ‘postnatal support’ OR ‘postnatal-support’ OR ‘postnatal support service*’ OR ‘perinatal support’ OR ‘lay support*’ OR ‘health visitor*’ OR ‘buddy support’ OR ‘budd* project’) **OR AB** (‘peer support*’ OR ‘peer-support*’ OR ‘peer group*’ OR ‘social support*’ OR ‘support group*’ OR ‘community support*’ OR ‘peer counsel*’ OR ‘peer mentor*’ OR ‘peer assist*’ OR ‘mother-to-mother support’ OR ‘one-to-one peer support’ OR ‘individual peer support’ OR ‘peer-led support’ OR ‘supportive interaction*’ OR ‘peer educator’ OR ‘peer listener’ OR ‘peer advocate’ OR ‘postpartum support’ OR ‘postnatal support’ OR ‘postnatal-support’ OR ‘postnatal support service*’ OR ‘perinatal support’ OR ‘lay support*’ OR ‘health visitor*’ OR ‘buddy support’ OR ‘budd* project’)
**S8 (String 8/Keywords S7)**	(‘Peer Counsel*’ OR ‘Peer Group*’ OR ‘Social Support*’ OR ‘Community Support*’ OR ‘Support Group*’)
**S9 (String 9)**	S1 OR S2
**S10 (String 10)**	S3 OR S4
**S11 (String 11)**	S5 OR S6
**S12 (String 12)**	S7 OR S8
**S13 (String 13)**	S9 AND S10 AND S11 AND S12

### Study selection

Results were exported to ‘Zotero’ and then uploaded to ‘Covidence’, a systematic review management tool, to facilitate and streamline the screening process. Following the removal of duplicates, the remaining papers were primarily screened based on their titles and abstracts by the lead author. Although standard practice for scoping and systematic reviews typically involves two independent reviewers at both the title and abstract and full-text screening stages, due to resource constraints and the defined scope of this project, a single-reviewer approach was employed for the initial title/abstract stage. Subsequently, a comprehensive full-text screening process was conducted by three authors (KT, ÁOC and EJOS). Furthermore, to ensure a comprehensive identification of relevant studies, reference list searching was performed. This involved screening the reference lists of papers included to identify additional sources that may have been overlooked in the database search. After examining these references, no additional studies met the inclusion criteria or were deemed suitable for inclusion in the review.

**Figure 1.**
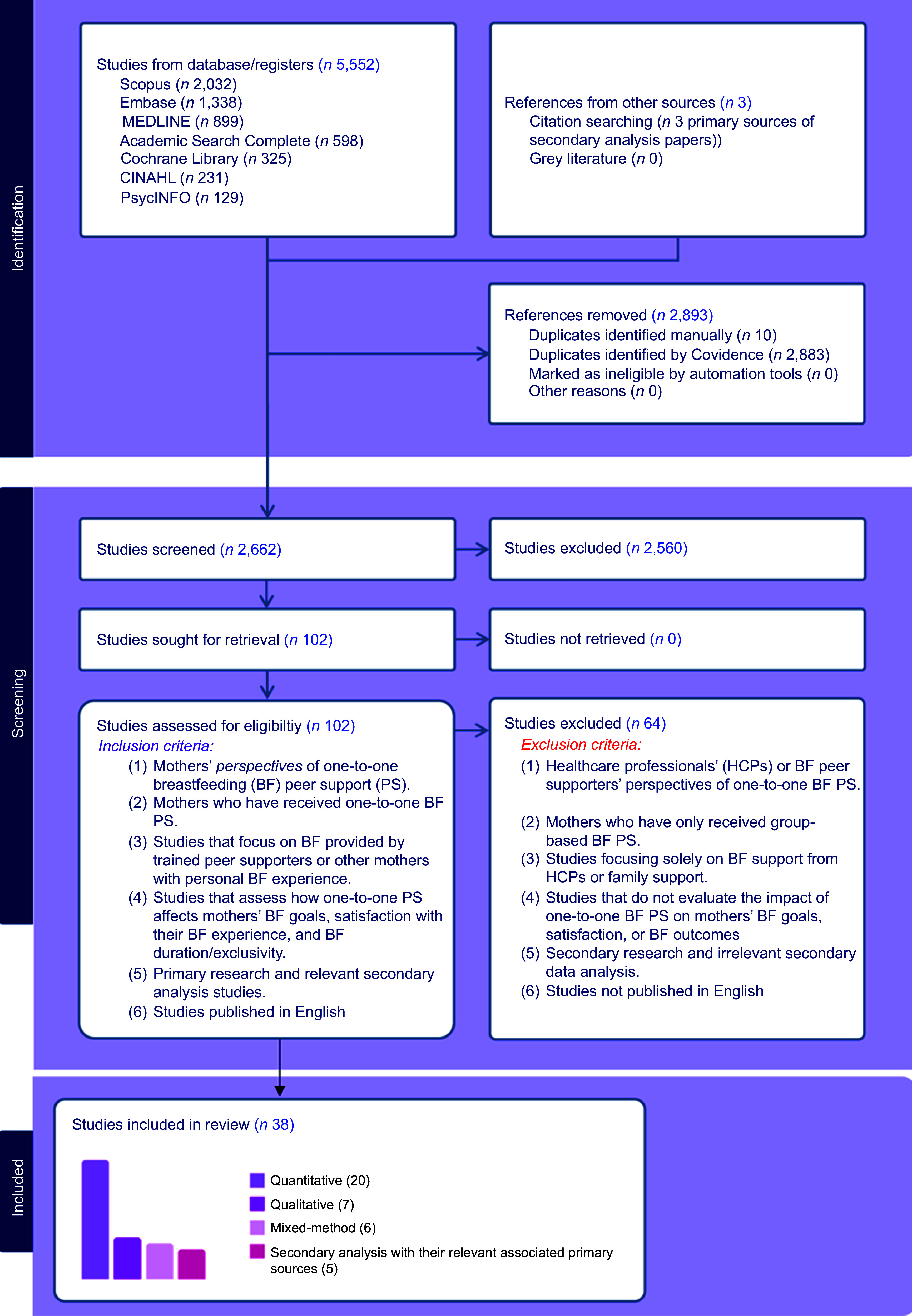
PRISMA flow diagram of search process^(26)^

Papers were included if they described the mothers’ experience/perspective of one-to-one peer breastfeeding support. Papers were excluded if they focused on group breastfeeding support or support delivered solely by family members or healthcare professionals such as midwives, lactation consultants and general practitioners. The inclusion and exclusion criteria that guided this review are detailed in Table 3. During the full-text screening stage, the lead author initially assessed each paper independently. Papers with uncertain eligibility were further reviewed by a second and third reviewer, who initially assessed papers for inclusion independently and then met to discuss. A consensus was reached on the inclusion or exclusion of studies, with any disagreements resolved through discussion. To enhance the clarity of the review process, the review was documented using the PRISMA-ScR flow diagram (Figure 1)^(27)^. A diverse range of studies were chosen to be included, i.e. qualitative, quantitative, relevant secondary data analysis and mixed methods to capture both numerical and thematic insights.

**Table 3.** Inclusion/exclusion criteria Table 3 long description.

Inclusion criteria	Exclusion criteria
Mothers’ perspectives of one-to-one peer breastfeeding support.	Healthcare professionals’ or breastfeeding peer supporters’ perspectives of one-to-one peer breastfeeding support.
Mothers who have received one-to-one peer breastfeeding support.	Mothers who have only received group-based peer breastfeeding support.
Studies that focus on breastfeeding support provided by trained peer supporters or other mothers with personal breastfeeding experience.	Studies focusing solely on breastfeeding support from healthcare professionals (e.g. midwives, lactation consultants) or family support (e.g. partner)
Studies that assess how one-to-one peer support affects mothers’ personal breastfeeding goals, satisfaction with their breastfeeding experience and breastfeeding outcomes.	Studies that do not evaluate the impact of one-to-one peer breastfeeding support on mothers’ personal breastfeeding goals, satisfaction or breastfeeding outcomes.
Primary research: Qualitative, quantitative and mixed method studies (e.g. interviews, focus groups, surveys, randomised control trail, cohort studies). Relevant secondary analysis studies.	Secondary research: Systematic reviews, meta-analyses, literature reviews, scoping reviews, editorials, conference abstracts and discussion papers. Irrelevant secondary data analysis.
Studies published in English.	Studies published in languages other than English.

### Charting the data

This component of the scoping review involved extracting/charting key items of information obtained from the papers reviewed^(27)^. Each included paper was analysed by two reviewers to minimise bias and ensure reliability and consistency of findings. Arksey and O’Malley’s^(27)^ process was utilised to extract the required data from each paper, including author, year, title, country, aim, methodology/study design, sample size, description of the interventions, summary of findings and considerations. Any questions about included/excluded data were resolved through discussions among the review team.

### Collating, summarising and reporting the results

In accordance with the Joanna Briggs Institute Manual for Evidence Synthesis^(31)^ and PRISMA-ScR guidelines^(26)^, data from the included studies were collated and summarised using descriptive methods appropriate for scoping reviews. Quantitative data (e.g. intervention characteristics and breastfeeding outcomes) were summarised using frequency counts and proportions^(31)^. Qualitative data were analysed using descriptive content analysis^(31)^. Extracted findings and participant quotations were read repeatedly to familiarise reviewers with mothers’ experiences of one-to-one breastfeeding PS. Line-by-line coding identified key concepts, including personal breastfeeding goals, perseverance through challenges, the importance of encouragement, emotional reassurance and mother-centred support. These codes were grouped into meaningful categories reflecting the ways in which one-to-one PS influenced mothers’ personal breastfeeding goals, including how support helped mothers set, achieve or extend their personal goals, overcome challenges and build confidence on their breastfeeding journey. Tables and figures were used to present study characteristics (Table 4) and a description of interventions employed in the included studies (Table 5), providing a clear visual overview of the literature.

**Table 4.** Data extraction table Table 4 long description.

Author/s, year, title, country	Aim/focus of paper	Methodology/Design	Sample size & population	Data collection/Analytical approach	Summary of findings	Considerations
Agrasada *et al.*, 2005 Postnatal peer counselling on exclusive breastfeeding of low-birthweight infants: a randomised controlled trial. Philippines.	Test the efficacy of postnatal peer counselling among first-time mothers that aimed to increase exclusive breastfeeding of term LBW infants.	Quantitative, randomised control trail (RCT).	Mothers (*n* 204): BFPC home visits (*n* 68); childcare home visits (*n* 67); control group (CG) no home visits (*n* 69). Completed 6-month trial (*n* 179).	Structured interviews (recall-based questionnaire) & clinical assessments by physicians.	Mothers who received breastfeeding peer counselling (BFPC) were 6·3 times more likely to EBF from 2 weeks-6 months. At 6 months, EBF rates during the previous 7 d were 44 % (BFPC), 7 % (childcare) and 0 % (control); any breastfeeding rates were 63·2 %, 31·3 % and 29 %, respectively. Considering continuous EBF from birth to 6 months, 32 % of mothers in the BFPC group maintained EBF throughout, compared with 3 % (childcare group) and 0 % (control group). Mothers valued the support and viewed BFPC as key to their success.	BFPS is an essential component of BF promotion programmes and may be useful when integrated into the primary care level of healthcare services. Important that HCP and peer supporters have defined roles and collaborate to achieve common BF goals. BFPS is a successful intervention aiming to achieve EBF among term LBW infants.
Anderson *et al.*, 2005 A randomised trial assessing the efficacy of peer counselling on exclusive breastfeeding in a predominantly Latina low-income community. United States of America.	To assess the efficacy of peer counselling to promote exclusive breastfeeding (EBF) among low-income inner-city women in Hartford, Conn.	Quantitative RCT.	Mothers (*n* 135).	Baseline interview; second interview & review of medical records during postpartum hospital stay. Follow-up interviews via phone weekly (month 1), biweekly (months 2–3). Statistical analysis (SPSS).	PC group 15 times more likely to EBF compared to CG. At hospital discharge, 24 % of CG had not initiated BF, compared to 9 % in PC group. PC coverage highest during the prenatal period. 88·9 % of mothers received a home visit. PC coverage decreased to 63·5 % by 6 weeks postpartum. Prenatal home visits averaged 2·6 ± 1·9 h. In-hospital PS sessions averaged 2·2 ± 2·0 h.	BFPC highly effective in promoting EBF among communities with exceedingly low rates of EBF and strong preference toward feeding formula even if they choose to breastfeed their infants. Strong potential for promoting EBF among low-income women through peer counselling in the United States.
Battersby and Sabin, 2001 Breastfeeding peer support – ‘the Worldly-Wise Project’ United Kingdom.	To develop and evaluate a new model of peer support for breastfeeding, particularly targeting mothers in socially disadvantaged areas.	Mixed Method.	Mothers (*n* 16).	Interviewed (*n* 5), questionnaire (*n* 11).	BFPS encouraged mothers to BF for longer. BFPS visits varied postnatally from 1–6 visits or more. All but one mother found PS to be very helpful, informative and supportive.	Mothers should be offered flexible support options (home visits, phone contact) based on their needs. Antenatal breastfeeding workshops should be developed to improve knowledge and confidence. BF PC should deliver these supports to reduce conflicting advice.
Burns *et al.*, 2020 Breastfeeding support at an Australian Breastfeeding Association drop-in service: a descriptive survey. Australia.	To examine women’s experiences of accessing one breastfeeding drop-in peer support service provided by trained peer support volunteer counsellors from the ABA.	Mixed Method.	Mothers (*n* 51).	Open-ended qualitative survey. Thematic analysis Braun & Clarke, 2006. Quantitative survey data analysed, descriptive statistics.	90 % of women, reported that the drop-in PS service helped them to achieve their BF goals. Beyond providing practical and informational assistance, PS offered significant emotional support. Non-judgmental, mother-focused approach was deeply valued. Most women used the drop-in service when infants were 0–8 weeks (72 %), nearly half attended within the first 4 weeks (49 %).	Low-cost, in-person emotional and practical BF support for mothers may help identify BF challenges earlier and contribute to longer BF durations.
Chapman, Damio, & Pérez-Escamilla., 2004 Differential response to breastfeeding peer counselling within a low-income, predominantly Latina population. United States of America.	To identify those most responsive to breastfeeding peer counselling (PC) using data from a US-based randomised trial.	Quantitative, RCT.	Mothers (*n* 165)	Monthly phone interviews over 6 months to collect demographic and feeding data. Medical records reviewed. Multivariate logistic regression analyses.	BF behaviours different in maternal subgroups, i.e. Primiparous *v*. Multiparous. At 3 & 6 months postpartum, adjusted EBF rates among PC group were 57·3 % and 32·9 %, respectively, compared to 3- and 6-month BF rates of 43·9 % and 29·9 %, respectively, for the CG.	Antenatal contact with a PC is essential for multiparae and for those who have not yet decided to BF. Multiparae women may have experienced prior breastfeeding challenges or negative experiences and benefit from reassurance that these difficulties can be managed. BFPC provides education, emotional support and practical strategies, bolstering confidence and motivation to initiate breastfeeding. It can also help mothers navigate logistical barriers, such as caring for older children, reinforcing that breastfeeding is feasible alongside other responsibilities.
Chapman *et al.*, 2004 Effectiveness of breastfeeding peer counselling in a low-income, predominantly Latina population: a randomised controlled trial. United States of America	To evaluate the effectiveness of an existing, breastfeeding peer counselling programme within the United States.	Quantitative, Randomised prospective controlled trial.	Mothers (*n* 165)	Initial interview. Follow-up interviews via phone until BF stopped and up until 6 months. Data analysis: SPSS software.	Mothers in the intervention group (IG) had a 61 % lower relative risk of not initiating BF compared to the CG. The IG demonstrated a 28 % and 22 % lower relative risk of not BF at 1 month and 3 months, compared to CG. At 1 month postpartum, 35·7 % of the IG were not BF *v*. 49·3 % of CG. At 3 months, 55·6 % of the IG were not BF *v*. 70·8 % of CG. All participants in the IG received at least one PC contact.	BFPC positively impacts BF outcomes in low-income populations. Well trained PC, working under the supervision of IBCLC, can make a difference in BF practices. PC considered to be a valued part of medical team. PC understand the socio-economic and cultural barriers of BF women and empower mothers to overcome challenges.
Chiwara and Villate, 2013 (Primary source of: Saniel *et al.*, 2021) Final evaluation of the joint Programme: ensuring food security and nutrition for children 0–24 months in the Philippines (MDG-F 2030) Philippines.	To contribute to the improvement of nutritional status of 0–2-year-old children and complement government’s efforts through social marketing strategies to increase the percent of exclusive breastfeeding through nationwide efforts.	Mixed-method summative programme evaluation – reporting on quantitative findings.	Not specified	The findings from this report are the primary source for the secondary analysis of Saniel *et al.*, 2021	PC were effective in promoting EBF. However, 20–30 % of trained volunteers were inactive, and 20 % reported limited understanding of their roles. Delays in developing training materials postponed peer group formation and pushed intensive PC training to late 2011.	PC found to be a sustainable strategy for EBF and was very likely to be continued because the activities were implemented through existing structures and systems of the government’s localised health care delivery system.
Clark *et al.*, 2018 Breastfeeding peer support programme increases breastfeeding duration rates among middle- to high-income women. United States of America.	To determine whether a breastfeeding peer support programme would increase breastfeeding duration rates among middle-to high-socio-economic status women.	Quantitative, prospective cohort study.	Mothers (*n* 113)	Observational data collected through prenatal visits, phone calls. Anticipatory BF guidance provided throughout the infants’ first year of life. Data analysis: SPSS.	85 % of the women at 6 months and 65 % at 12 months who remained in the Omega Smart Baby Project for BFPS were successfully BF, compared to Colorado statewide EBF rates of 56 % at 6 months and 27 % at 12 months. Reasons for BF cessation included perceived low milk supply, personal BF goals were met, issues latching baby, returning to work, maternal and infant health concerns, baby self-weaned or new pregnancy. The BFPS programme included guidance before birth and throughout the programme.	Consistent BF support during the 1st year of life has the potential to increase BF duration rates for women of higher socio-economic status.
Cueva *et al.*, 2017 Strengths and Challenges of the Alaska WIC Breastfeeding Peer Counsellor Programme: a Qualitative Study of Programme Implementation. United States of America.	To explore the implementation of a breastfeeding peer counsellor programme with Alaska *Special Supplement Nutrition Programme for Women, Infants and Children (WIC)*.	Mixed Method.	WIC staff and BFPC (*n* 33) interviewed, WIC clients (BF mothers): focus groups (*n* 25) and surveyed (*n* 129).	In-depth interviews, 5 focus groups and surveys. Data analysis: Content analysis. Atlas.ti.	BFPC helped mothers BF for longer. Interactions with BFPC viewed as overwhelmingly positive. Social support identified as a contributor to BF success. Participants valued BFPCs’ support, especially for isolated women with limited social networks. BFPC were knowledgeable and accessible to support women with challenges. BFPC provided practical and informational support. information and advice on BF latch etc. Interaction with BFPC varied. About 40 % of women at 1 site indicated that they had seen a BFPC just once, whereas about half at another site shared that they had seen a BFPC ≥ 3 times.	Texting and online platforms offered ongoing BF support, bridging gaps in communication and access. PC could relate to the clients as they often shared similar cultural or socio-economic backgrounds. Challenges included BFPCs’ limited hours, funding and in-person contact with clients and confusion about the BFPCs’ role.
Dennis, 2002 Breastfeeding peer support: maternal and volunteer perceptions from a randomised controlled trial. Canada.	To describe maternal and peer volunteer perceptions of their experience while participating in a breastfeeding peer support trial.	Quantitative, RCT.	Primiparous BF women (*n* 258): PS group (*n* 132), CG (*n* 126). Peer volunteers (*n* 58); 30 completed questionnaires; 78 submitted activity logs (reflecting multiple logs submitted by some volunteers who supported more than one mother).	Mothers’ perception of PS Questionnaire (*n* 130). Data analysis: Descriptive statistics and content analysis.	More PS group mothers maintained EBF at 3 months and reported higher feeding satisfaction. Mean PS duration was 53·1 d; interactions averaged 16·2 min. 96 % of initial contacts occurred in week 1; 67 % within 48 h post-discharge, 30 % before discharge. 71 % received ≥ 2 contacts in week 1, 46·2 % in week 2. 35·5 % of PS relationships ended by month 2, 64·5 % continued monthly and 19·7 % extended past 3 months. 85 % said they would have a peer volunteer again; all felt PS should be offered to new mothers. 65 % said PS helped them meet BF goals; 14 % didn’t have any difficulties didn’t require assistance. Reasons for discontinuing PS early: no support needed (40 %), stopped BF (27 %), no relationship formed (27 %), or support declined (6 %).	Mothers and peer volunteers reported positive experiences and high satisfaction. Most mothers felt they had enough contact and support during challenges. Peer volunteers suggested improvements such as ongoing training, peer networking opportunities, recognition of their contributions and confirming that enrolled mothers genuinely wanted support.
Dennis, 1999 A randomised controlled trial evaluating the effect of peer (mother-to-mother) support on breastfeeding duration among primiparous women. Canada.	To evaluate the effect of peer support on breastfeeding duration among first-time mothers.	Quantitative, RCT (Part of Thesis).	PS group (*n* 132). CG (*n* 124).	Questionnaire Data analysis: Descriptive statistics, SPSS.	More women in the PS group decided to BF before pregnancy compared to the CG. More women in the PS group EBF at 4 and 12 weeks postpartum. Satisfaction with PS was high, 62 % very satisfied. 85 % of mothers would have a peer volunteer again. PS valued for different reasons: informational (18·1 %), emotional (3·1 %), appraisal (6·3 %). 14 % appreciated talking to an experienced mom. 9·4 % liked knowing help was available. 7 % felt PS was especially helpful for those without nearby family. Of the (14·6 %) who would not want PS again, (8·5 %) had enough support, (2·3 %) were dissatisfied. All participants agreed that every new mother should have access to PS. 70·5 % of mothers didn’t feel PS affected BF duration, 20 mothers credited the volunteer with helping them persevere.	Telephone-based PS, when combined with HCP services and existing social networks, could be an effective clinical intervention to increase the number of primiparous women who continue BF until 3 months postpartum. Peer volunteers may be effective mediating links between mothers in the community and HCP.
Forster *et al.*, 2019 Proactive Peer (Mother-to-Mother) Breastfeeding Support by Telephone (Ringing up About Breastfeeding Early [RUBY]): A Multicentre, Unblinded, Randomised Controlled Trial. Australia.	To determine whether proactive telephone-based peer support during the postnatal period increases the proportion of infants being breastfed at six months of age.	Quantitative, RCT.	Women randomly assigned to peer support (*n* 574) or usual care (*n* 578).	Questionnaire. Data analysis: intention to treat in Stata Version 14.	More infants of women assigned to proactive telephone-based PS were receiving any breast milk at six months (75 %) *v*. (69 %) assigned to usual care. Women in the PS group had a (23 %) lower risk of stopping BF than the usual care group. Mothers were linked with PC about 3·2 d postpartum, 85 % matched within 4 d. Initial phone contact was around day 7. Each mother received about 6 calls. Among those supported > 4 weeks (64 %), median calls reached 11 over 20+ weeks. One-third had support for 26 weeks; 36 % ended before 4 weeks.	Providing first-time mothers with telephone-based PS with at least six months personal BF experience is an effective intervention for increasing BF continuation in settings with high BF initiation rates.
Fox *et al.*, 2015 UK women’s experiences of breastfeeding and additional breastfeeding support: a qualitative study of Baby Café services. United Kingdom.	To explore the qualitative experiences of UK mothers who used Baby Café services, with a focus on their experiences of breastfeeding and the support they received.	Qualitative: In-depth interviews and focus groups.	Primiparous women (*n* 33) and multiparous women (*n* 18). Total (***n* 51**) mothers.	36 in-depth interviews during BF support sessions and 5 focus groups at the Baby Café session. Data analysis: NVivo software, thematic analysis.	Mothers valued expert and social support at Baby Cafés. PC seen as BF role models. Cafés ran weekly, staffed by professionals, trained peers and volunteers. Women valued the face-to-face contacts with skilled BF professionals. For some, visiting the Baby Café was seen as a ‘turning point’ in their BF relationship. Women valued a BF peer supporter with an ‘authentic presence’ where a trusting relationship or rapport with the supporter could be built. PC ensured needs were met, provided an empathetic and caring approach and affirmation of the mother’s own abilities. Ongoing support was seen as vital for meeting or extending BF goals amid challenges like weaning or returning to work.	The need for realistic rather than idealistic antenatal preparation and the importance of timely and parent-centred BF support, particularly in the immediate postnatal weeks highlighted. Effective social support, combined with reassurance and guidance from skilled practitioners, can help women to overcome difficulties and find confidence in their own abilities to achieve their feeding goals. Further work needed to ensure such services are readily accessible to all women.
Gonzalez-Darias *et al.*, 2020 ‘Supporting a first-time mother’: Assessment of success of a breastfeeding promotion programme. Spain.	To measure the effectiveness on breastfeeding rates by the programme ‘Supporting a First-time Mother’, a web-based platform of interaction between first-time mothers and breastfeeding-experienced women who act as peer supporters.	Quantitative, RCT.	Intervention group (*n* 76); control group (*n* 78). Total mothers: (*n* 154)	Questionnaire. Research team monitored participants access to the website and its content, as well as participant-supporter interactions. Data analysis: statistical analysis using SPSS software.	76 % of mothers EBF at 3 months in PS group *v*. 56 % (CG). At 6 months, (60 %) of mothers were EBF in the PS group *v*. 44 % (CG). BFPS increases the success of BF by 2·65 times exactly on average. Success with BF was greater when mothers had a higher educational level. Mothers who engaged with PS were more likely to still be BF at 3 months compared to those who didn’t make contact. 94·4 % of mothers who contacted their peer supporter were still BF at 3 months.	Programme has been shown to help increase BF rates in first-time mothers by offering one-to-one support from experienced volunteers through low-cost technology. Making this programme more accessible, especially to young mothers or those with less education could further improve BF rates.
Graffy *et al.*, 2004 Randomised controlled trial of support from volunteer counsellors for mothers considering breastfeeding. United Kingdom.	To investigate whether offering volunteer support from counsellors in breastfeeding would result in more women breastfeeding.	Quantitative, RCT.	Women initially recruited (*n* 720) – intervention group (*n* 363), control group (*n* 357).	Questionnaires. Statistical analysis – SPSS and STATA software.	80 % of the women in the IG received an antenatal BFPS contact. Postnatally the counsellors visited 20 % of the women at least once, spoke with 43 % by telephone and had no contact with 38 %. Women who left school earlier were less likely to arrange visits. Initial BF rates were 95 % (IG) *v*. 96 % (CG) (*P* = 0·44); BF rates at 6 weeks, 65 % (IG) *v*. 63 % (CG) were BF (*P* = 0·69); BF rates at 4 months, 46 % (IG) *v*. 42 % (CG) (*P* = 0·33). BFPS were rated very helpful by 73 %. Free-text responses showed women valued the relationship, practical advice and increased BF knowledge.	Offering mothers additional voluntary BFPS did not extend the duration of BF perhaps because those who stopped were less likely to seek help or delay the introduction of artificial formula feeds.
Harari *et al.*, 2018 Feasibility and acceptability of a text message intervention used as an adjunct tool by WIC breastfeeding peer counsellors: The LATCH pilot. United States of America.	To test the acceptability and feasibility of the lactation advice through a texting intervention.	Mixed Method.	Texting intervention (*n* 30), CG (*n* 22). Total mothers: (*n* 52)	Follow-up telephone survey. Data analysis: quantitative data analysis (Stata 12.1); qualitative open-ended survey questions open-ended survey questions thematically analysed using both deductive and inductive elements (Crabtree & Miller, 1999).	Contact between mothers and PC within 48 h of delivery was significantly higher in the texting group (86·6 %) compared to the control group (27·3 %, *P* < 0·001). At 2 weeks the EBF rate was 50 % (IG) *v*. 31·8 % (CG). Mothers in the IG were meeting their BF goals more frequently (96·1 %) *v*. CG (73·6 %). 91 % of mothers in the texting group would recommend this support to a friend. They felt the intervention improved breastfeeding knowledge, prepared them for breastfeeding, offered needed support, eased communication through texting and allowed them to seek advice anytime.	Breastfeeding education and PS via two-way text messaging is very feasible among WIC participants. Consistent text messaging helped to foster the perception of constant support and the ability for mothers to reach out for help and advice.
Ingram *et al.*, 2013 A mixed methods evaluation of peer support in Bristol, UK: mothers’, midwives‘ and peer supporters’ views and the effects on breastfeeding. United Kingdom.	To evaluate mothers’, midwives’ and peer supporters’ views of peer support and the effects on breastfeeding.	Mixed Method.	Mothers completed online survey (*n* 163); 25 participants were interviewed mothers (*n* 14), peer supporters (*n* 7) and maternity health professionals (*n* 4)	Surveys, semi-structured interviews and focus groups. Data analysis: thematic analysis using an inductive approach	96 % reported that they had received an antenatal visit from their peer supporter. After the initial postnatal call, 31 % stayed in touch for advice. Antenatal visits were described as helpful, encouraging and clear Postnatal calls were seen as supportive and reassuring. 91 % felt the timing of the first postnatal contact was appropriate; 7 % said it came too late. Initial postnatal contact was often via text, followed by calls or visits if BF support was needed. Mothers valued the reassurance and advice, which boosted confidence and helped them continue BF. PS was viewed as essential for all mothers. Those with an antenatal PS visit averaged six contacts in the first two postnatal weeks. At 48 h, 70 % EBF and 19 % partially BF. By two weeks, 54 % EBF and 17 % partially BF (71 % total BF).	Continuity of PS with an antenatal visit and postnatal support from the same peer supporter was thought to be beneficial.
Ingram *et al.*, 2020 Women’s and peer supporters’ experiences of an assets-based peer support intervention for increasing breastfeeding initiation and continuation: A qualitative study. United Kingdom.	To explore women’s and infant feeding helpers’ (IFH) views of the different components of the assets-based feeding help before and after birth (ABA) intervention.	Qualitative, descriptive study.	Mothers (*n* 21) IFH (*n* 13)	Semi-structured interviews and focus groups. Data analysis: thematic analysis, NVivo.	Assets-based approaches focus on positive capabilities of individuals and communities, rather than their needs, deficits and problems. Early face-to-face antenatal contact-built trust and encouraged ongoing communication. Peer supporters used proactive texting and took a woman-centred, not breastfeeding-centred, approach. Support was kind, reassuring and non-judgmental. Mothers felt supported when not pressured to breastfeed and when given encouragement and clear direction to appropriate help.	This proactive, community-based, woman-centred approach was well received by both mothers and peer supporters, showing promise as an intervention that requires further research on its impact on infant feeding outcomes.
Jolly *et al.*, 2012 Effect of a peer support service on breastfeeding continuation in the UK: A randomised controlled trial. United Kingdom.	To assess the effectiveness of a peer support worker (PSW) service on breastfeeding continuation.	Quantitative, cluster randomised controlled trial.	Total enrolled: 2724 mothers. Randomised to IG (*n* 1267); CG (*n* 1457). Consented to 6-month follow-up (*n* 848).	Routine computerised records and Questionnaire at 6 months. Statistical analysis = intention to treat.	No difference in any breastfeeding at 10–14 d between groups. Initiation rates: 89·7 % (IG) *v*. 88·0 % (CG). Any breastfeeding at 6 weeks: 62·7 % (IG) *v*. 64·5 % (CG); at 6 months: 34·3 % (IG) *v*. 38·9 % (CG). PS was offered to all of the IG; 80 % had contact. Postnatal PS was provided to those who initiated BF (747/1140 births). 61·6 % received postnatal PS; 58·8 % had contact within a week, 48·4 % had a second session. Sessions averaged 28 min. Among BF mothers, 75 % had PS within 48 h of discharge; 86 % within 72 h.	Universal antenatal and postnatal PS for women who initiated BF did not improve BF rates up to 6 months in this UK population. A higher uptake of BFPS might have led to a more positive effect on BF rates.
Kabakian-Khasholian *et al.*, 2019 Experiences with peer support for breastfeeding in Beirut, Lebanon: A qualitative study. Lebanon.	To describe the experiences of breastfeeding mothers and peer support providers with the process of breastfeeding support and the influence of the intervention on their social support system	Qualitative study (cross-sectional study).	Mothers (*n* 22) Peer supporters (*n* 21)	In-depth interviews. Data analysis: thematic analysis.	Peer supporters offered moral support and encouragement during BF challenges. One mother described PS as ‘the push you need to continue.’ Peer supporters shared their own BF experiences which helped mothers feel reassured and realise that difficulties are normal and shared by many. BF mothers became advocates by talking with other women in their social circles sharing information, correcting myths and offering BF tips.	PS systems influence more than BF outcomes, as they provide a structure for building a community of BF advocates. Creating mother-to-mother support groups may be a cost-effective way to help improve BF rates in similar communities.
Knox *et al.*, 2023 Text message conversations between peer supporters and women to deliver infant feeding support using behaviour change techniques: A qualitative analysis. United Kingdom.	To analyse text message conversations between peer supporters (called Infant Feeding Helpers – IFH) and new mothers using qualitative methods to understand how peer support can influence and support women’s feeding experiences.	Qualitative analysis study.	Primiparous women (*n* 18) and Infant Feeding Helpers (*n* 7).	Antenatal meeting recordings, semi-structured interviews and text message analysis. Data analysis: Inductive thematic analysis (Braun and Clarke 2006) of the text messages in NVivo. Deductive content analysis of the antenatal meetings, interviews and text messages.	Of the 18 mothers, 7 planned to EBF, 7 to mostly BF and 4 did not intend to BF. By 8 weeks, 12 were EBF or mixed feeding. IFH checked in daily for the first 2 weeks; texting continued for an average of 6 months (range 2·5–9). Mothers often re-initiated contact for support with common issues (e.g. nipple pain, unsettled babies). IFH provided practical strategies, emotional support and personalised, friendly conversations. They supported all feeding choices. Mothers valued help most in the first 2 weeks, especially listening, encouragement, BF groups, online resources and links to wider support.	IFH effectively provided engaging infant feeding PS via text. Findings suggest text messaging is an acceptable and useful method for delivering PS, especially when in-person support is limited due to unforeseen circumstances, such as the COVID-19 pandemic.
Linares *et al.*, 2019 Las Dos Cosas *v*. Exclusive Breastfeeding: A Culturally and Linguistically Exploratory Intervention Study in Hispanic Mothers Living in Kentucky. United States of America.	To assess the feasibility, effectiveness and acceptability of a culturally and linguistically diverse intervention to promote exclusive breastfeeding (EBF) for the first 6 months.	Quantitative, Exploratory RCT.	39 Hispanic pregnant women recruited and randomly assigned to intervention (*n* 20) and control groups (*n* 19).	Surveys. Data analysis: descriptive and inferential statistical analysis	More mothers in the PS group EBF (45 %) *v*. CG (21 %) during their hospital stay. Over time, those in the PS group were about 3 times more likely to continue EBF compared to those without extra support. Intervention involved PC and IBCLC providing BF support through prenatal and postpartum visits and follow-up calls. 93 % of the IG were satisfied or very satisfied with the programme. 70 % received 2 prenatal home visits; 30 % received 1. 70 % received planned hospital visit. IBCLC visited 75 % at home; PC visited 45 %. 60 % had ≥ 2 prenatal follow-up calls; others had 1. 75 % received 2 postnatal follow-up calls. Missed sessions were due to missed/unanswered calls. Attrition was 30 %, mostly by the 6-month follow-up.	Culturally and linguistically tailored intervention was well accepted by Hispanic mothers, helped increase EBF duration and reduce early formula supplementation.
Lok *et al.*, 2021 Feasibility, acceptability and potential efficacy of an innovative postnatal home-based breastfeeding peer support programme in Hong Kong: a feasibility and pilot randomised controlled trial. Hong Kong.	To determine the feasibility of a breastfeeding support programme with home-based visits from peer supporters over a six-month period among postpartum Chinese women in Hong Kong.	Mixed Method.	BFPS programme: (*n* 10) CG: (*n* 10)	Questionnaire and interviews. Data analysis: descriptive statistical analysis and thematic analysis of interviews.	Most women planned to EBF: 90 % (IG) and 80 % (CG). 7 IG and 8 CG participants aimed to EBF for 6 months. Home-based PS visits were valued for BF information, emotional support and boosting confidence. Mothers appreciated accessible advice and encouragement, describing PC as knowledgeable and approachable. Participants averaged 2·2 PS sessions. WhatsApp was used for ongoing communication when visits weren’t possible. Mothers suggested starting PS earlier in pregnancy, integrating it into antenatal classes and providing more guidance around 6 months, especially on complementary feeding.	Recruiting and training existing peer supporters was feasible, and they were able to deliver the intervention in a way that was acceptable to women. PS seen as a financially sustainable solution to provide home-based BF PS.
MacArthur *et al.*, 2009 Antenatal peer support workers and initiation of breastfeeding: cluster randomised controlled trial. United Kingdom.	To assess the effectiveness of an antenatal service using community-based breastfeeding peer support workers on initiation of breastfeeding.	Quantitative, cluster RCT.	Total pregnant women from 66 clinics (*n* 2511); 33 clinics received PS intervention (*n* 1140), 33 clinics received standard care (*n* 1371).	Hospital records. Statistical analysis – intention to treat.	Initiation rates for BF did not differ between IG (69·0 %) and CG (68·1 %). The mean duration of the first support session was 13·1 min, and 94·4 % took place in the clinic, with only 1·3 % at home.	The antenatal PS service had no effect on breastfeeding initiation in a diverse and socio-economically deprived population.
Martinez *et al.*, 2020 ‘Real-world’ effect of a peer counsellor on breastfeeding outcomes in an urban prenatal clinic in the United States. United States of America	To assess the ‘real life’ effects of an autonomous peer counsellor who provides tailored support to low-income, minority women based on individual needs rather than a predetermined research protocol.	Secondary analysis of a prospective cohort study (Navigating New Motherhood, NNM); quantitative. The primary source does not discuss BF PS.	Women enrolled in NNM study (*n* 218)	Data analysis: statistical bivariable analysis and multivariable logistic regression models.	Women in the PC cohort were more likely to initiate BF (89·8 %) *v*. those without a PC (78·9 %, *P* = 0·04). No significant differences were observed in BF continuation at 6 weeks (55·6 % PC *v*. 49·1 % no PC) or 6 months postpartum (21·8 % *v*. 18 %). Significantly higher satisfaction with BF training was reported in the PC cohort during postpartum hospitalisation (98·2 % *v*. 83·6 %, *P* = 0·006), however this equalised by 6 weeks.	Peer counsellor interventions show potential for improving BF initiation rates.
McCoy *et al.*, 2017 Associations between peer counselling and breastfeeding initiation and duration: An analysis of Minnesota participants in the special supplemental nutrition programme for women, infants and children (WIC). United States of America.	To identify associations between Minnesota WIC peer breastfeeding support programme services and breastfeeding initiation and continuation.	Quantitative, Retrospective analysis of observational data.	Mothers (*n* 2219)	Data analysis: statistical analysis	Odds of BF initiation were significantly higher among those who received PS prenatally (1900 initiated BF) *v*. those without PS. Women who received PS had a significantly lower risk of BF discontinuation than women who did not receive services from month one right through to month twelve.	Access to PS was linked to higher BF initiation and continuation up to 12 months. Expanding these services could be especially beneficial in areas with low BF rates.
McInnes *et al.*, 2000 Evaluation of a community-based intervention to increase breastfeeding prevalence. United Kingdom.	To determine whether peer counselling in the antenatal and postnatal period would increase the prevalence and duration of breastfeeding among low-income women in Glasgow.	Quantitative, quasi-experimental evaluation.	Women enrolled (*n* 995): IG (*n* 474), CG (*n* 521). Data to 6 weeks postnatal (*n* 919).	Questionnaires. Statistical analysis – multivariate analysis, intention to treat.	At booking, 18 % (IG) and 21 % (CG) intended to BF. At delivery, the proportions initiating BF were 23 % (IG) and 20 % (CG), and by 6 weeks postnatally, the proportion providing any breastmilk had declined to 10 % (IG) and 8 % (CG). By 6 weeks postnatally the difference between the two groups was not statistically significant (*P* = 0·07).	Only 70 % of the IG received at least one PS antenatal visit, therefore the impact of the intervention may have been diluted. A more structured PS intervention may be preferable to overcome the reluctance of mothers to spontaneously request PS.
McLardie-Hore *et al.*, 2020 Proactive telephone-based peer support for breastfeeding: a cross-sectional survey of women’s experiences of receiving support in the RUBY randomised controlled trial. Australia.	To explore women’s experiences of receiving the RUBY peer support intervention.	Quantitative, RCT – cross-sectional survey.	Women respondents to survey (*n* 360)	Survey. Data analysis: statistical data analysis, content analysis used for short answered open-ended survey responses, long open-ended survey responses thematically analysed.	Ringing Up about Breastfeeding earlY (RUBY). 79 % of women found telephone PS ‘helpful’ to ‘very helpful’. 93 % satisfied; 89 % would recommend. Mothers valued empathy, encouragement and non-judgmental support from peer supporters with BF experience. PS offered emotional reassurance, practical guidance and boosted confidence, sometimes extending BF goals. Telephone support was praised for accessibility, flexibility, continuity and personalised contact. 95 % received at least one contact; frequency and duration varied by need.	Accessible, proactive telephone BFPS is vital. Women valued the information and emotional support (empathy, reassurance, encouragement) provided by their peer supporter. Flexible call scheduling and combining texts with calls tailored to mothers’ needs is recommended to enhance PS.
Meier *et al.*, 2007 A qualitative evaluation of a breastfeeding peer counsellor programme. United States of America	To identify the programme’s strengths, operation procedures and improvement areas from participants’ and peer counsellors’ perspectives.	Qualitative, descriptive study.	Mothers (*n* 20)	Focus group interviews. Data analysis: content analysis following Krueger & Casey, (2000) framework.	The PC programme provided emotional support, BF guidance and resource access (e.g. pumps). Most had personal BF goals, often set collaboratively with their PC, which motivated them to continue. Antenatal attendees felt well-prepared for BF. Strong, trusting PC-mother relationships formed, with many viewing PC as family-like advocates. Many women described their PC as a family-like figure/personal advocate. Length and frequency of visits varied based on individual needs. Trust, empathy and approachability were key to the programme’s success.	The peer counsellor model can effectively support low-income BF women.
M’Liria *et al.*, 2020 Impact of Mother-to-Mother Support Groups in Promoting Exclusive Breastfeeding in a Low-Resource Rural Community in Kenya: A Randomised Controlled Trial. Kenya.	To assess the impact of community-based MTMSG with or without income-generating activities in promoting EBF in low socio-economic rural setting in Kenya.	Quantitative, RCT.	Mothers (*n* 249)	Questionnaire Data analysis: statistical data analysis SPSS software.	CG = routine postnatal care. Treatment Group 1 (MES): Monthly mother-to-mother support groups (MTMSG) with structured, facilitated discussions focused on EBF and related practices, held at local health facilities. Treatment Group 2 (MESIGA): Followed the same MTMSG model as treatment group 1 but included an additional income-generating activity session after each BF support meeting. The MESIGA group showed consistently higher EBF rates each month from birth to 6 months, compared to the CG. At 1 month postpartum, mothers in the MESIGA group were over twice as likely to EBF compared to the CG. EBF rates gradually declined across all groups over time. MESIGA and MES groups maintained significantly higher EBF rates than the CG at every follow-up, up to 6 months. MESIGA group EBF for an average of 3·4 months, compared to 2·8 months in MES group and just 0·7 months in the CG. 27 % of mothers in MESIGA EBF for six months, compared to 10 % in MES and 0 % in CG.	Mother-to-mother support groups are an effective approach to promoting EBF in low-income rural areas and should be expanded in Kenya and similar contexts.
Monoto *et al.*, 2020 Breastfeeding peer counsellor programme in Malaysia: Impact on breastfeeding duration and exclusivity. Malaysia.	To explore the effect of peer counselling support on breastfeeding outcomes among postnatal mothers who delivered at a national tertiary teaching hospital and their satisfaction with the peer counselling services.	Quantitative, Single-blinded RCT.	Total postpartum mothers (*n* 210): IG (*n* 105); CG (*n* 105).	Semi-structured, closed-ended questionnaires via telephone interview. Data analysis: SPSS statistical analysis.	EBF was higher in the IG than the CG at all intervals with a significant difference seen at 6 months (*P* = 0·035). More participants in the IG EBF up to 6 months (46·7 % *v*. 32·1 %). 76·7 % found that the PC helped them to achieve their BF goals. 88·7 % of the participants would recommend PC services to others. 87·7 % of the participants regardless if they were EBF or not were satisfied with their interaction with the PC.	PC support in Malaysia increased the duration and exclusivity of BF and was well accepted among postnatal mothers.
Rozga *et al.*, 2014 Impact of peer counselling breastfeeding support programme protocols on any and exclusive breastfeeding discontinuation in low-income women. United States of America.	To describe associations between programme components (individual and combinations) and breastfeeding outcomes (duration and exclusivity) in a PC programme for low-income women.	Quantitative, observational cohort study (secondary analysis of routine programme data).	Women enrolled prenatally (*n* 5886)	Demographic data collected at enrolment. At each postnatal visit the PC recorded the mothers infant feeding status. Data analysis: statistical analysis via Stata software	92·2 % of women in PS programme-initiated BF. Average BF duration: 21·3 weeks; 77 % BF at 1 month, 55 % at 3 months, 40 % at 6 months, 26 % at 1 year. EBF initiation 78 %, mean EBF duration 11·1 weeks; 60 % EBF at 1 month, 6 % at 6 months. Each additional PS contact (home, phone, other) reduced risk of discontinuing both any and exclusive BF. Participants received an average of 9 PS contacts (3 home visits, 5 calls, 1 other). They were enrolled in the programme for around 31 weeks, with 21 of those weeks occurring postnatally.	In-person contact with trained peer counsellors can positively influence BF continuation. Combining in-person and phone support may further enhance outcomes, potentially mitigating maternal and infant risk factors for early discontinuation.
Olson *et al.*, 2010 (Primary Source of: Rozga *et al.*, 2014) A quasi-experimental evaluation of a breastfeeding support programme for low-income women in Michigan. United States of America.	To examine the effectiveness of a peer counselling breastfeeding support programme for low-income women in Michigan who participate in WIC.	Quantitative, Quasi-Experimental Evaluation.	Total mothers (*n* 990): IG (*n* 336); CG (*n* 654).	Data derived from administrative and survey-based sources. Data analysis: statistical analysis via STATA software.	The Breastfeeding Initiative (BFI) is a collaboration between the Michigan’s Women, Children and Infant (WIC) Programme and Michigan State University Extension. The programme significantly improved BF outcomes. Mothers in the IG breastfed on average 2·6–3·6 weeks longer than those in the CG. They were also (22·3 %) more likely to initiate BF, (9·0 %) more likely to continue at 3 months and (6·2 %) more likely at 6 months.	BFPS programme effectively increased BF rates among low-income women, a population that’s BF rates are well below the national average and below what is recommended by public health professionals. BFI programme warrants a thorough cost-benefit analysis to assess its cost-effectiveness and should be tested in other settings to determine if its positive outcomes can be replicated.
Saniel *et al.*, 2021 Effectiveness of peer counselling and membership in breastfeeding support groups in promoting optimal breastfeeding behaviours in the Philippines. Philippines.	To determine the effectiveness of visits by peer counsellors during pregnancy and after delivery and membership in breastfeeding support groups in promoting these optimal breastfeeding practices.	Quantitative, secondary analysis (cross-sectional) of Endline Survey data. Before-and-after non-experimental (quasi-experimental) study).	Mothers (*n* 2343)	Data from the Endline Survey ‘Millennium Development Goals – Fund 2030 Joint Programme (JP) on Ensuring Food Security and Nutrition for Children 0–24 months old’. Data analysis: logistic regression methods.	During pregnancy, 23·5 % had a PC visit and 33·4 % joined a BF support group. Both were linked to early BF initiation. PC visits prenatally and postnatally increased EBF odds by 15 % and 28 %, respectively, but not significantly. BF support group participation significantly increased EBF odds.	BF support groups could be formally integrated into the healthcare system to encourage both early initiation and EBF in the. The role of peer counsellors in supporting optimal BF practices warrants further evaluation.
Scott *et al.*, 2017 The impact of breastfeeding peer support for mothers aged under 25: a time series analysis. United Kingdom.	To evaluate the effectiveness of a breastfeeding peer support service (BPSS) in one UK city in increasing breastfeeding initiation and duration in young mothers.	Quantitative, time series statistical analysis.	Mothers (*n* 5790)	Health visitors collected infant feeding data during routine home visits. At the two- and six-week visits, they observed and recorded current feeding practices. Data analysis: Statistical analysis via STATA software.	Breastfeeding Peer Support Service (BPSS). 45 women accessed the Nottingham BPSS from October 2012 to September 2013, representing 29 % of the eligible population. The proportion of eligible women accessing BPSS increased over time, from 4 % in October 2012 to 61 % by September 2013. Each participant had a median of 6 contacts. Pre-BPSS Trends: BF at birth and two weeks: No significant month-to-month change before the BPSS introduction. BF at 6 weeks: increase of 0·09 % per month prior to BPSS. Post-BPSS introduction: BF rates increased: At birth: increase of 0·55 % per month. At 2 weeks: increase of 0·50 % per month. By the end of the study, 6·6 more women per 100 were initiating BF each month. 6 more women per 100 BF at two weeks compared to pre-intervention rates. No change at 6 weeks in BF prevalence post-BPSS.	One-to-one BPSS provided by paid peer supporters and targeted at young mothers in the antenatal and postnatal periods may be beneficial in increasing BF initiation and prevalence at 2 weeks.
Srinivas *et al.*, 2015 A Clinic-Based Breastfeeding Peer Counsellor Intervention in an Urban, Low-Income Population: Interaction with Breastfeeding Attitude. United States of America.	To improve rates of any and exclusive breastfeeding at 1 and 6 months using a low-intensity peer counselling intervention beginning prenatally and to study the interaction of breastfeeding attitude and self-efficacy with the intervention.	Quantitative, RCT.	Mothers (*n* 103): IG: (*n* 50); CG (*n* 53).	Validated survey instruments: (BSES-SF) and (IIFAS). Data analysis: descriptive and inferential statistical analysis.	66 % of participants had prenatal PC contacts, 85 % occurred by telephone. Of the 43 participants who initiated breastfeeding, (95 %) had at least 1 contact, and (30 %) had at least the number of PC contacts specified by the protocol for their duration of breastfeeding. Median duration of BF was 6 weeks (IG group = 7·0 weeks, CG = 6·0 weeks). No difference between the IG and CG BF initiation (IG = 86 % *v*. CG = 77 %, *P* = 0·34), EBF before hospital discharge (44 % CG *v*. 40 % CG, *P* = 0·95), or BF to 1 month (68 % IG *v*. 53 % CG, *P* = 0·14) or 6 months (4 % in each group). Women in the IG tended to achieve their personal BF goal, but this difference did not achieve statistical significance (43 % *v*. 22 %, *P* = 0·073). Significantly more women in the IG felt that BFPS was very respectful (IG = 73 %, CG = 24 %; *P* < 0·001). Significantly fewer women in the IG felt that standard care was sufficient (IG = 18 %, CG = 61 %; *P* < 0·004).	BF rates increased across all participants during the study period. BF attitudes showed a stronger correlation with BF behaviour than PS. PS was beneficial for women with low self-efficacy and supported them in reaching their BF goals.
Tan *et al.*, 2017 Using the Solihull Approach in breastfeeding support groups: Maternal perceptions. United Kingdom.	To formally explore maternal perceptions of this peer support breastfeeding service.	Qualitative, semi-structured interviews. Observational data.	Mothers (*n* 9)	Semi-structured interviews and observational data collection. Data analysis: thematic analysis	BF cafes, run by midwives, trained peer breastfeeding supporters and community volunteers. BF cafes were described as ‘inviting’ and ‘welcoming’. Staff and volunteers at the BF cafes were seen as available, reliable and trustworthy. Some mothers built strong bonds with specific staff, especially community volunteers who showed unconditional positive regard for mothers. Informational support is what mothers were seeking when they first attended the BF cafes. The quality of the information at the BF cafés was said to surpass that of HCP. The BF cafes were a form of emotional support. Mothers could discuss their personal difficulties with their peer supporter and receive tailored advice for their needs. Emotional support provided in the form of encouragement, reassurance and verbal and non-verbal expressions of caring.	This study demonstrated that this method of BF support can provide consistent, personalised informational support and responsive emotional, benefiting mothers from diverse socio-economic backgrounds.
Thomson *et al.*, 2012 Giving me hope: women’s reflections on a breastfeeding peer support service. United Kingdom.	To explore health professionals’, peer supporters’ and women’s experiences, facilitators, barriers and challenges faced in the introduction of a breastfeeding peer support service.	Qualitative exploratory evaluation.	Total women: (*n* 47)	Focus groups and in-depth interviews. Face-to-face (*n* 30), telephone interview (*n* 17). Data analysis: thematic analysis, MAXQDA software.	Star Buddies fostered hope through realistic assessments provided across varying situational contexts and scenarios. They helped women modify their strategies. Star Buddies shared their own and others’ experiences of BF, providing BF women with a range of goal-directed thoughts and strategies. Women’s specific BF goals were fluidly determined (e.g. weekly) with some hoping to EBF for 6 months. Star Buddies directly influenced BF targets through consistent contact with women. Support and guidance often enabled women to extend and enhance their BF goals. Women referenced to PS as a ‘lifeline’, ‘safety net’. As women knew where they were able to access trusted help and support, this often motivated the woman to continue BF. Enhanced women’s confidence, skills and self-beliefs.	PS can foster hope and motivation in BF women through strategies such as individualised planning, continuous feedback, flexible and accessible support, and encouragement of social connections. Integration of realistic goal-setting, regular reassessment and positive reinforcement may enhance the effectiveness of BF support services.

**Table 5.** Description of interventions of included studies Table 5 long description.

Author/s	Single-component intervention (one-to-one peer support)	Multi-component intervention (one-to-one peer support plus additional healthcare professional support)
Agrasada *et al.*, 2005	**Intervention:** *Breastfeeding counselling* – Peer counsellors provided education on exclusive breastfeeding up to 6 months and support to prevent and manage breastfeeding problems, delivered through eight scheduled home visits (days 3–5, 7–10, 21; 1·5 months; then monthly to 5·5 months). *Childcare counselling* – Peer counsellors provided infant care guidance and promoted mother–infant interaction (e.g. infant massage, smile therapy) through the same eight scheduled home visits as above. **Control group:** Usual postnatal care with no additional counselling.	*No additional professional lactation support was provided.*
Anderson *et al.*, 2005	**Intervention group:** One-to-one breastfeeding peer support in addition to routine maternity care. Trained peer counsellors provided 3 antenatal home visits, daily in-hospital visits following birth and up to 9 postnatal home visits over the first 6 weeks postpartum, with additional telephone support available as needed. Support focused on exclusive breastfeeding education, practical breastfeeding assistance and responsive, needs-led counselling. **Control group:** Mothers received usual care within a Baby-Friendly Hospital setting, including routine antenatal breastfeeding education, hands-on breastfeeding support from maternity ward staff after birth and access to on-call lactation consultants if clinically indicated. *Peer counsellors were trained and supervised by an IBCLC; mothers did not receive direct IBCLC support as part of the intervention*.	*No additional professional lactation support was provided.*
Battersby and Sabin, 2001	One-to-one breastfeeding peer support intervention delivered in a socio-economically disadvantaged area. Local women with personal breastfeeding experience were recruited and trained through the La Leche League Peer Counselling Programme. Peer support workers provided antenatal and postnatal breastfeeding support via home visits, telephone contact and attendance at antenatal clinics. Support was informal, relational and focused on encouraging and sustaining breastfeeding through shared lived experience, alongside existing midwifery and health visiting services. *The total duration of the intervention was not stated*.	*No additional professional lactation support was provided.*
Burns *et al.*, 2020	Australian Breastfeeding Association (ABA) peer support drop-in service offered ad hoc, face-to-face support one day per week. ABA primarily supports mothers via a 24-hour telephone helpline, online platforms and community group meetings. *The total duration of the intervention was not stated*.	*No additional professional lactation support was provided.*
Chapman, Damio, & Pérez-Escamilla., 2004	**Intervention:** Routine breastfeeding education (as per control) plus one-to-one peer counselling: 1 prenatal home visit, daily in-hospital postpartum visits, 3 postnatal home visits and monthly telephone follow-up for 6 months. Peer counsellors were trained mothers providing individualised breastfeeding support. **Control:** Routine breastfeeding education provided by clinic staff during prenatal visits; hands-on breastfeeding assistance from midwife’s post-birth; access to hospital lactation consultant on request; standard postnatal care.	*No additional professional lactation support was provided.*
Chapman *et al.*, 2004	**Intervention:** Routine care plus structured one-to-one peer counselling from trained mothers with personal breastfeeding experience. Intervention included at least 1 prenatal home visit, daily in-hospital visits during postpartum stay, 3 postnatal home visits and monthly telephone follow-up for 6 months. **Control:** Routine prenatal breastfeeding education, hands-on breastfeeding assistance from a midwife post-birth and access to the hospital’s lactation consultant as needed for breastfeeding problems.	*No additional professional lactation support was provided.*
Chiwara and Villate, 2013	As part of the Millennium Development Goals Achievement Fund 2030 Joint Programme on Ensuring Food Security and Nutrition for Children 0–24 Months in the Philippines, key activities were implemented to promote exclusive breastfeeding through community-based support strategies. Peer counsellors engaged with pregnant and postpartum mothers to encourage early initiation of breastfeeding and exclusive breastfeeding, complementing broader behaviour-change communication activities across programme sites in urban and rural areas. Peer counsellor visits were delivered alongside other programme activities focused on infant and young child feeding and nutrition. *The total duration of the intervention was not stated*.	*No additional professional lactation support was provided.*
Clark *et al.*, 2018	One-to-one peer support programme providing prenatal and postnatal breastfeeding guidance via scheduled in-person visits (hospital and home visits at 2, 4, 6 and 10 months) and phone calls (2 weeks, 1 month, 6 weeks, 3, 5, 7, 8, 9, 11 and 12 months), with additional support provided as needed. Each contact included an individualised assessment of the mother’s breastfeeding needs, with continued support up to 12 months postpartum. Peer counsellors had access to a lactation professional for guidance if required.	*No additional professional lactation support was provided.*
Cueva *et al.*, 2017	Individualised one-to-one peer support through in-person visits, phone calls, texting and online support groups. Contact frequency varied, with most support offered in the first weeks postpartum (2–3 contacts per week during the first month). *The total duration of the intervention was not stated*.	*No additional professional lactation support was provided.*
Dennis, 2002	**Intervention:** Received routine postpartum care plus one-to-one peer support. Telephone-based support was initiated within 48 h of hospital discharge, with some contacts made before discharge. Two or more peer support contacts in the first week and ongoing support up to 3 months postpartum. Mean duration of support: 53 d. Mean duration of telephone support call: 16 min. 97 % received telephone support and 3 % received face-to-face support. **Control:** Received routine postnatal care. No peer support was offered.	*No additional professional lactation support was provided.*
Dennis, 1999	**Intervention:** Predominantly telephone-based one-to-one support (97 % phone, 3 % face-to-face). Average contact duration: 16 min. Mean mother/peer supporter relationship: 23 d. Most mothers received ≥ 2 peer support contacts in the first 2 weeks; contact frequency decreased over 3 months, with 19·7 % continuing past 3 months. **Control:** Received routine postnatal care. No peer support was offered.	*No additional professional lactation support was provided.*
Forster *et al.*, 2019	**Intervention:** Routine postnatal care plus proactive one-to-one support. Peer supporters initiated telephone contact 24–48 h after hospital discharge. Mothers then received a follow-up call 3–4 d later, weekly calls for the first 12 weeks and calls every 3–4 weeks up to six months. **Control:** Routine postnatal hospital stay (48–72 h) with access to lactation consultants, 1–2 home midwife visits in the first week, ongoing Maternal & Child Health Nurse support and access to Australian Breastfeeding Association 24/7 telephone helpline. No peer support offered.	*No additional professional lactation support was provided.*
Fox *et al.*, 2015	N/A	Baby Café breastfeeding support groups providing face-to-face, drop-in support in community settings. Support was delivered through a social model combining informal peer (mother-to-mother) support with direct access to trained facilitators, including midwives, health visitors, breastfeeding counsellors and lactation consultants. Sessions ran weekly, were free of charge and women could attend as often and for as long as needed. Support included emotional reassurance, practical breastfeeding assistance and assessment by healthcare professionals when required.
Gonzalez-Darias *et al.*, 2020	**Intervention:** Routine postnatal care plus participation in the ‘Supporting a First-time Mother’ programme. Mothers received 24-hour access to an online breastfeeding support platform offering evidence-based information and one-to-one peer support from an assigned supporter with breastfeeding experience. Support was delivered exclusively online, allowed anonymous contact and continued for up to 6 months postpartum. Peer supporters responded to breastfeeding queries, provided reassurance and guidance, and recommended mothers contact health professionals when needed. **Control:** Routine postnatal care provided by hospital and community health services, including standard breastfeeding support from health professionals. This could include midwife or clinic support and optional attendance at breastfeeding support groups, with no structured peer support or online programme provided.	*No additional professional lactation support was provided.*
Graffy *et al.*, 2004	**Intervention:** Routine postnatal care plus one-to-one breastfeeding peer counsellor support (National Childbirth Trust). The intervention involved 1 antenatal home visit and postnatal support offered on request, delivered by telephone and/or additional home visits. Counsellors used a non-directive, confidence-building approach and provided practical breastfeeding advice, written information and emotional support. The duration and intensity of postnatal support varied by participant uptake, with support available up to four months postpartum. **Control:** Routine antenatal and postnatal care provided by general practices and maternity services, including breastfeeding advice from health professionals. No peer support intervention offered.	*No additional professional lactation support was provided.*
Harari *et al.*, 2018	N/A	**Intervention:** In addition to usual WIC breastfeeding peer counselling, participants received a two-way text-messaging intervention initiated antenatally (18–30 weeks’ gestation) through the early postpartum period. The intervention included automated, personalised breastfeeding education texts and the ability to exchange messages directly with a peer counsellor. The intervention was delivered throughout pregnancy, and peer counsellors contacted mothers within 48 h of birth. Text-based breastfeeding support continued for the first two weeks postpartum. The intervention was primarily one-to-one peer support, with additional guidance from healthcare professionals if complex breastfeeding challenges arose. **Control:** Usual WIC breastfeeding peer counsellor support delivered through standard programme contacts, without use of text messaging or additional peer supporter digital communication.
Ingram *et al.*, 2013	N/A	Multi-component intervention providing one-to-one peer support to pregnant women in areas with low breastfeeding rates. The peer supporter conducted a home visit around 32 weeks’ gestation to discuss infant feeding choices, involving partners or family where relevant, followed by a phone contact 48 h after hospital discharge. Text message support continued for two weeks postpartum. The peer supporters worked alongside midwives and maternity support workers, and breastfeeding peer counsellors were also available to mothers through attending support groups.
Ingram *et al.*, 2020	Trained Infant Feeding Helpers (IFH) provided one-to-one support to first-time mothers using a woman-centred, assets-based approach. Support began antenatally with a face-to-face meeting to discuss infant feeding, map social networks via an Infant Feeding Genogram and identify community assets. Postnatal support was offered daily for the first 2 weeks, then less frequently up to 5 months, delivered through a combination of face-to-face visits, phone calls, and text messages. The intervention was proactive (IFH initiated contact), inclusive of all infant feeding intentions, and used behaviour change strategies to provide social support. Text messaging was the most preferred and convenient mode of support for mothers.	*No additional professional lactation support was provided.*
Jolly *et al.*, 2012	**Intervention:** One-to-one peer support service offered antenatally and postnatally in addition to routine maternity care. Antenatal support aimed to provide two sessions, with at least one in the mother’s home, though most were delivered in the antenatal clinic or Children’s Centre. Postnatal support was needs-based, beginning with contact within 24–48 h of hospital discharge for women who initiated breastfeeding, with at least one further contact during the first week and additional follow-up by telephone or home visits as required. Each peer support session lasted an average of 28 min. **Control:** Routine maternity care received, including standard antenatal and postnatal midwife support, in-hospital breastfeeding advice and routine health visitor contact. No peer support intervention offered.	*No additional professional lactation support was provided.*
Kabakian-Khasholian *et al.*, 2019	N/A	**Intervention:** Participants received a multi-component breastfeeding support programme that included prenatal education delivered by lactation consultants, postpartum professional lactation consultant support through home visits and mainly telephone-based one-to-one support. Peer support followed a minimum schedule of ten contacts, beginning with the antenatal class (6–9 months gestation), contact during the expected week of birth, on the first day postpartum, 48 h after hospital discharge, at one, two and four weeks postpartum and monthly thereafter up to 6 months, with flexibility to adjust based on the mother’s needs. Lactation consultant support began on the first postpartum day in the hospital and continued daily (15–30 min) until discharge, followed by home visits on days 1, 3, 7, 15 and then monthly until 6 months, breastfeeding discontinuation, or maternal request. Support was primarily face-to-face but could also be delivered via telephone, with additional visits permitted if requested by the mother or deemed necessary by the lactation consultant. **Control:** Participants received standard postpartum care with no additional lactation consultant or peer support beyond routine hospital and community services.
Knox *et al.*, 2023	**Intervention:** ABA (Assets-based feeding help before and after birth) Infant Feeding Helpers (peer supporters) provided woman-centred support via face-to-face antenatal meetings and daily text messages for the first two weeks postpartum. Peer counsellors provided support up to five months postpartum. Support included social, emotional and practical guidance using Behaviour Change Techniques. **Control:** Routine maternity care received (infant feeding support from midwives, health visitors and community breastfeeding groups). No peer support intervention offered.	*No additional professional lactation support was provided.*
Linares *et al.*, 2019	N/A	**Intervention:** Mothers received a culturally and linguistically tailored breastfeeding support intervention led by a bilingual lactation consultant and a trained peer counsellor from the local Hispanic community. The intervention offered 1–2 antenatal visits, one in-hospital visit (post-birth), two postpartum home visits and pre/postnatal follow-up phone calls as needed. Informational materials, action plans and motivational strategies were provided to raise awareness, address barriers and promote exclusive breastfeeding for six months. Support was flexible to accommodate mothers’ schedules and continued until six months postpartum. **Control:** Routine maternity care. Both groups delivered in a Baby-Friendly hospital with access to hospital lactation consultation support. Control participants had no contact with the study lactation consultant or peer counsellor team.
Lok *et al.*, 2021	**Intervention:** Mothers received home-based breastfeeding one-to-one peer support which included five scheduled home visits: two in the first month postpartum (first week and third week) and three additional visits at 2, 4 and 6 months postpartum. Each visit lasted 30–60 min. Peer supporters initiated contact with mothers via phone within the first 48 h postpartum and maintained telephone contact between visits as needed. Support was continued until the mothers decided to stop breastfeeding. **Control:** Mothers received standard antenatal and postpartum care with no additional home-based peer support. Standard care included routine support from healthcare providers.	*No additional professional lactation support was provided.*
MacArthur *et al.*, 2009	**Intervention:** Community-based antenatal breastfeeding peer support programme delivered in addition to routine midwifery care. Trained peer supporters were matched, where possible, to women by ethnicity and language. Support included an initial introduction followed by at least two planned antenatal contacts at approximately 24–28 and around 36 weeks’ gestation, with at least one home visit. The duration and content of peer support sessions were tailored to women’s needs. Peer supporters provided culturally appropriate information, addressed concerns and barriers to breastfeeding and offered postnatal follow-up to women who initiated breastfeeding. **Control:** Routine maternity care. No additional community-based peer support intervention offered.	*No additional professional lactation support was provided.*
Martinez *et al.*, 2020	**Intervention:** Routine maternity care plus individualised breastfeeding peer support. The peer supporter provided regular antenatal contact with mothers (2–3 contacts in the third trimester), an in-hospital postpartum visit and ongoing postpartum support delivered flexibly via clinic visits, inpatient contact and text messaging, according to the mother’s needs. *The total duration of the intervention was not stated*. **Control:** Routine maternity care received. No peer support intervention offered.	*No additional professional lactation support was provided.*
McCoy *et al.*, 2017	Pregnant women were assigned a trained peer counsellor. Peer support was delivered primarily via telephone, with some text, email or in-person contacts. Support began during pregnancy and continued through to 12 months postpartum, depending on the mother’s needs. Peer counsellors provided breastfeeding education and encouragement and recommended that the mothers refer to a lactation consultant for complex breastfeeding issues.	*No additional professional lactation support was provided.*
McInnes *et al.*, 2000	**Intervention:** Routine maternity care plus community-based one-to-one peer support programme including a minimum of four contacts: two antenatal visits (in the second and third trimester) focused on infant feeding decision-making and breastfeeding education, and at least two postnatal contacts providing practical, individualised breastfeeding support. Postnatal support was flexible and responsive to the mothers’ needs, with women who initiated breastfeeding receiving a mean of four postnatal visits (range 1–15 visits). **Control:** Routine maternity care received. No peer support intervention offered.	*No additional professional lactation support was provided.*
McLardie-Hore *et al.*, 2020	**Intervention:** Routine maternity care plus proactive one-to-one support primarily via telephone, beginning shortly after birth and continuing for up to 6 months postpartum. Support was typically weekly in the first 3 months, then reduced in frequency (fortnightly to monthly) thereafter, with calls lasting around 6–20 min. Additional peer support was available by text message as needed, and the content was tailored to the mother’s individual breastfeeding needs. **Control:** Routine maternity care received. No peer support intervention offered.	*No additional professional lactation support was provided.*
Meier *et al.*, 2007	A Mother-to-Mother breastfeeding peer counsellor programme targeting low-income women enrolled in the Women, Infants, and Children (WIC) programme. One-to-one support beginning in pregnancy and continuing up to 12 months postpartum or until breastfeeding cessation. Support included at least one antenatal home visit, monthly antenatal phone calls, a hospital visit when feasible, a phone call within 48 h of delivery, one or more early postpartum home visits, weekly phone calls during the first postpartum month and monthly phone calls thereafter. Additional contacts were provided as needed. Support was primarily delivered via home visits and telephone contact, with content focused on breastfeeding education, encouragement, problem-solving and emotional support.	*No additional professional lactation support was provided.*
M’Liria *et al.*, 2020	**Intervention 1: Mother-to-mother support group with education and support (MES)**: Support included one antenatal session and six-monthly postnatal group sessions over approximately six months. Sessions lasted about one hour, were facilitated by trained peer supporters and focused on exclusive breastfeeding education, practical skills (positioning and attachment) and management of common breastfeeding challenges. Informal support from peers and facilitators was available between sessions. **Intervention 2: Mother-to-mother support group with education and support plus income-generating activities (MESIGA)**. Women received the same Mother-to-Mother Support Group breastfeeding peer support as intervention group 1. In addition, participants took part in a one-hour income-generating activity after each group meeting from 2 to 7 months postpartum, intended to improve attendance and sustained participation. **Control:** Routine maternity care received plus irregular group health education sessions delivered at antenatal and maternal-child health clinics, with limited breastfeeding information. No peer support intervention offered.	*No additional professional lactation support was provided.*
Monoto *et al.*, 2020	**Intervention:** The breastfeeding one-to-one support intervention commenced within 72 h of hospital discharge, with scheduled telephone calls at 2 weeks and at 1, 2, 4 and 6 months postpartum (minimum of six contacts). Additional calls were provided as needed. Support continued until 6 months postpartum, withdrawal from the study, or cessation of breastfeeding. **Control:** Routine maternity care received. No peer support intervention offered.	*No additional professional lactation support was provided.*
Olson *et al.*, 2010	**Intervention:** One-to-one peer support intervention including at least one in-person contact, followed by monthly contacts via telephone or in-person based on maternal needs. On average, mothers received 3 home visits, 2 personal contacts outside the home and 6 telephone contacts over approximately 24 weeks. Women remained in the programme until they discontinued breastfeeding, the infant turned 1 year old or support was no longer needed. **Control:** Routine maternity care received. No peer support intervention offered.	*No additional professional lactation support was provided.*
Rozga *et al.*, 2014	One-to-one peer support intervention providing breastfeeding education and assistance to low-income women. On average, participants received 2–3 home visits, 5 phone calls and 1 other contact, with support continuing until breastfeeding cessation, programme withdrawal or when the infant turned 1 year old. Additional ‘other’ peer supporter contacts, including texts, mail, or clinic visits, were provided as needed.	*No additional professional lactation support was provided.*
Saniel *et al.*, 2021	The Joint Programme intervention in the Philippines was a one-to-one peer support initiative aimed at improving breastfeeding practices among low-income mothers. The intervention included home visits by trained peer counsellors during the antenatal and postnatal periods, with additional follow-up via telephone contacts as needed to provide ongoing support. Peer counsellors offered individualised guidance, education, and encouragement on early initiation of breastfeeding and exclusive breastfeeding for six months. Mothers were also encouraged to participate in breastfeeding support groups during pregnancy, which provided additional social and informational support.	*No additional professional lactation support was provided.*
Scott *et al.*, 2017	The Nottingham Breastfeeding Peer Support Service was an intensive, one-to-one peer support intervention targeting mothers aged under 25 in a deprived urban area of the UK. Peer supporters provided individualised guidance from 30–34 weeks gestation until 6 weeks postpartum, with the highest intensity of support in the first two weeks postpartum. The intervention included proactive antenatal contact, home visits within 24–48 h of hospital discharge, and ongoing face-to-face, telephone, or text support relevant to each mother’s needs. The service was integrated with midwifery, health visiting, and children’s centres, and included regular supervision and access to professional consultation for peer supporters. On average, mothers received six contacts, comprising two face-to-face visits, two telephone calls and up to two text messages, ensuring continuous, tailored support during the early postpartum period.	*No additional professional lactation support was provided.*
Srinivas *et al.*, 2015	**Intervention:** One-to-one peer counselling initiated between 28 weeks’ gestation and one week before delivery, with additional contacts at the mother’s request. Postnatally, the peer counsellor followed up with mothers 3–5 d after birth, weekly to 1 month, every 2 weeks up to 3 months and once at 4 months, via clinic visits or telephone support. Home visits were generally not facilitated, except for maternal request in a few cases. **Control:** Received routine maternity care, which included hospital access to lactation consultants, outpatient lactation support and limited peer support availability (<once per month).	*No additional professional lactation support was provided.*
Tan *et al.*, 2017	N/A	The intervention consisted of drop-in breastfeeding cafés held four days per week. The cafés provided group-based peer support, with mothers able to attend for as long as they wished, ranging from the first week postpartum to several years. Mothers could receive individualised support from trained midwives, peer supporters or community volunteers during sessions or via phone and text. Support included tailored informational guidance on breastfeeding and practical breastfeeding support, as well as emotional support, such as reassurance, encouragement and help managing anxiety or stress. The approach created a safe, welcoming environment in which mothers felt accepted, listened to and empowered, fostering both improved breastfeeding practices and the desire to support other mothers.
Thomson *et al.*, 2012	The Star Buddies programme is a breastfeeding one-to-one support intervention delivered across the perinatal period. Support is provided by trained peer supporters through multiple modes, including one-to-one home visits, telephone calls, text messages, hospital bedside guidance, antenatal workshops and attendance at local breastfeeding groups. Peer support commences within 48 h post-discharge and continues for up to 8 weeks, with flexible, needs-based contact that mothers can extend as required.	*No additional professional lactation support was provided.*

### Findings

The search of the seven electronic databases and the addition of three primary source research papers generated 5555 results. After the removal of duplicates, 2662 remained and underwent title and abstract screening. At this stage, 2560 records were excluded because they did not meet the inclusion criteria, primarily because they were identified as irrelevant, did not address mothers’ experiences of one-to-one peer breastfeeding support or were review articles. The remaining 102 papers advanced to full-text review. Thirty-eight papers met the inclusion criteria, and justification for the excluded papers (*n* 64) is reported in the PRISMA flow diagram (Figure 1). The characteristics of the included papers, such as research methodology, year and country of publication, are detailed in Table 4.

### Characteristics of the studies

The thirty-eight papers included in this review were published between 1999 and 2023, spanning across ten countries globally (Table 4). Of the thirty-eight papers, twenty were quantitative studies^(15,16,32–49)^, seven were qualitative studies^(50–56)^ and six mixed methods^(57–62)^. An overview of the study designs employed in the included papers is provided in Table 4.

In this review, both the qualitative and quantitative findings from all mixed-methods studies were included. Three secondary analysis papers were also included, as they directly discussed one-to-one breastfeeding PS and addressed the research question^(63–65)^. Chiwara and Villate^(66)^, a mixed-methods study in which only the quantitative findings were reported, served as the primary source for the quantitative study by Saniel *et al.* ^(63)^. Martinez *et al.* ^(65)^, a quantitative study, was a secondary analysis of the ‘Navigating New Motherhood’ study^(67)^. Olson *et al.* ^(68),^ a quantitative study, was the primary source for the quantitative paper by Rozga *et al.* ^(64)^.

### Description of interventions

The included studies (*n* 38) employed a wide range of one-to-one breastfeeding support interventions, delivered using a combination of methods such as face-to-face, telephone and digital communication, employing both structured and flexible formats. Table 5 provides a detailed description of the intervention characteristics across included studies.

Interventions were broadly categorised as single- or multi-component. Among the thirty-eight included studies, 82 % (*n* 31) evaluated single-component one-to-one PS interventions, in which trained peer counsellors/supporters delivered one-to-one breastfeeding support alongside routine maternity care, without the addition of structured professional lactation input. In these interventions, peer supporters primarily provided emotional reassurance, practical breastfeeding guidance and encouragement, drawing on shared lived experience^(15,37,47,58)^. The remaining 18 % (*n* 7) evaluated multi-component interventions, in which one-to-one support was integrated with additional professional lactation consultant care, midwifery or health visitor input, structured infant care education or community-based services offering direct access to healthcare professionals^(40,49–51,54,60,61)^.

Across the thirty-eight included studies, the initiation of one-to-one support varied, with interventions commencing antenatally, postnatally or continuing throughout the perinatal period. Thirteen studies (34 %) initiated PS antenatally^(15,16,36–38,41,43,52,53,55,56,61,65)^, typically beginning between 24 and 28 weeks^(16)^ or around 30 weeks gestation^(53)^. These antenatal PS interactions were typically delivered through home visits or clinic-based meetings and focused on infant feeding decision-making and breastfeeding preparation^(16,40,44,45,48,61)^. Antenatal PS initiation was more frequently observed in interventions targeting first-time mothers or populations with historically low breastfeeding rates^(16,40,45,48,61)^. The majority of studies (66 %) initiated PS in the postnatal period, with first contact typically occurring within 24–72 h of hospital discharge, reflecting an emphasis on early breastfeeding support^(32,34–36)^.

The duration of PS offered widely varied, with 29 % (*n* 11) offering short-term support (≤8 weeks postpartum)^(15,32,34–36,39,53,55,57,60,61)^, 47 % (*n* 18) provided longer-term support (≥6 months or until breastfeeding cessation)^(16,33,40,41,43–50,56,59,63–65,68)^, and the remaining 24 % (*n* 9) did not clearly specify a fixed duration of support, instead reporting that contact frequency and continuation were determined by maternal needs^(37,42,51,54,56–58,62,66)^.

Face-to-face support, usually *via* home visits, was reported in 68 % of studies (*n* 26), ranging from 1–9 visits, with some daily in the early postpartum period^(15,16,33,35,37,39–41,43–47,51–53,56–59,61,62,64,65,68)^. Hospital-based support was included in 24 % of studies (*n* 9), providing bedside breastfeeding guidance^(40,42–46,48,49,65)^. Telephone support was reported in 71 % of studies (*n* 27), often frequent in the early postpartum period and reducing over time^(15,32–37,39,42–48,52,53,56,57,59–65,68)^. Digital support, including text messaging and online platforms, was reported in 29 % of studies (*n* 11), facilitating proactive, timely support^(15,42,46,51–54,56,59,60,62)^. Community-based PS support, such as drop-in cafés or peer-led clinics, were included in 16 % of studies (*n* 6), offering informal, flexible PS beyond the early postpartum period^(38,51,54,57,60,61)^.

PS was individualised and proactive, with frequency, duration and mode of delivery adjusted to meet mothers’ evolving needs, particularly during the early postpartum period, highlighting the flexibility and adaptability of one-to-one PS interventions.

The findings are presented according to the primary *a priori* outcome (the impact of one-to-one PS on mothers’ personal breastfeeding goals) and two secondary outcomes that emerged from the data, namely the effect of one-to-one PS on traditional metrics of breastfeeding success and the impact of mother-centred breastfeeding PS on maternal emotional well-being.

### Primary (a priori) outcome: Impact of one-to-one peer support on mothers’ personal breastfeeding goals

Twelve studies discussed the impact of PS on mothers’ personal breastfeeding goals, examining whether they were achieved or extended beyond their initial intentions^(33–35,39,48,51,55–58,60,62)^. Most mothers attending a drop-in PS service believed it helped them achieve their goals, with breastfeeding durations extending up to 21 months^(57)^. Similarly, another study reported that almost all mothers in the PS group met their personal breastfeeding goals compared to about three-quarters of the CG^(60)^. Another study emphasised that over three-quarters of mothers believed their peer counsellor helped them achieve their personal breastfeeding goals^(39)^. An additional study demonstrated that women receiving breastfeeding PS were more likely to achieve their personal breastfeeding goal, but this difference did not achieve statistical significance^(48)^.

Peer supporters not only helped women meet their initial goal but often encouraged mothers to extend their goals^(62)^. One mother shared: *‘I gave myself a 6-week goal and with all your encouragement…I’m almost to 8 months!’* ^(62)^. Furthermore, many mothers believed their peer supporter was instrumental in helping them reach their personal breastfeeding goals^(34,35)^. Twenty mothers described how their peer supporter helped them to ‘*persevere*’^(34,35)^. The importance of ongoing PS was crucial as it enabled women to meet or even extend their personal breastfeeding goals: *‘I would like to thank the volunteer mother…I may have stopped breastfeeding in the initial phases when everything felt too hard and overwhelming* ^(33)^ *.’*

Many women discussed how their peer supporter inspired them to create new personal breastfeeding goals by increasing their confidence*: ‘She helped me to feel proud and confident…achieving my goal of breastfeeding for 6–12 months’* ^(33)^. Another study found most women initially aimed to breastfeed for 3 months, then set new goals during each PS interaction^(56)^.

The ‘Star Buddies’ programme showed that consistent encouragement from peer supporters helped mothers extend their personal breastfeeding goals: ‘Just *knowing that they would be calling next week spurred me on’* ^(55)^. Similarly, another study highlighted that many mothers initially set short-term goals but continued breastfeeding much longer than expected due to PS: *‘I was looking at five weeks max…still breastfeeding at nine months’* ^(58)^.

These findings demonstrate the instrumental role one-to-one PS has in enabling mothers to achieve and often surpass their initial personal breastfeeding goals by offering reassurance, motivation, and practical guidance.

### Secondary outcome 1: The effect of one-to-one peer support on traditional metrics of breastfeeding success

Although this review focuses on one-to-one peer breastfeeding support and its impact on women’s personal breastfeeding goals, the literature predominantly reports outcomes in terms of initiation, duration and exclusivity of breastfeeding. These measures remain the primary indicators used by national and international bodies to define breastfeeding success. Mapping these outcomes provides essential context for understanding how PS has influenced both global breastfeeding targets and mothers’ personal goals. It also highlights a gap in the literature. While breastfeeding rates are well-documented, women’s individual goals remain underexplored. This gap helps justify the focus of this scoping review.

Nineteen of the included studies^(32,34–36,38–40,42–47,49,60,63–65,68)^ highlighted the positive effect of breastfeeding PS on increasing the initiation, duration and exclusivity of breastfeeding, with only eight papers reporting no significant differences in breastfeeding outcomes between the IG and CG^(15,16,37,41,48,59,63,65)^.

Mothers who received one-to-one breastfeeding PS were generally more likely to initiate and sustain EBF for longer compared to those without such support^(32,34,35,38–40,42–47,49,64,65,68)^. For instance, one study highlighted that women receiving PS were nearly three times more likely to EBF during their hospital stay^(49)^, while another found that mothers in the PS group were 15 times more likely to EBF compared to the CG throughout the study^(45)^. Another study reported higher EBF rates at six months among the PS group: 46·7 % (PS) *v*. 32·1 % (CG)^(39)^.

Furthermore, three studies highlighted significantly higher breastfeeding continuation rates among those who received PS at one, three and six months^(32,43,44)^. At one month postpartum, 64·3 % of the PS group were breastfeeding *v*. 50·7 % of the CG^(44)^. Similarly, another study demonstrated that mothers who received proactive telephone-based PS were more likely to breastfeed at six months: 75 % (PS) *v*. 69 % (CG)^(32)^. At three and six months postpartum, the EBF rates among the PS group were 57·3 % and 32·9 %, respectively, compared to 43·9 % and 29·9 % in the CG, respectively^(43)^. Structured interventions, like the ‘Omega Smart Baby Project’^(46)^, demonstrated increased breastfeeding duration and strong maternal satisfaction with PS^(33–35,37,39,40,50,51,54,56–60,65)^. Structured PS models like the mother-to-mother support groups with education and support (MES) and those with added income-generating activities (MESIGA) boosted EBF rates, with MESIGA achieving 27 % EBF at six months *v*. 0 % (CG)^(38)^. Mothers in the MESIGA programme were more than twice as likely to maintain EBF to six months compared to women receiving routine postnatal support, emphasising the added value of structured, consistent peer interactions^(38)^. Another study observed higher EBF rates at three and six months among mothers receiving PS^(42)^. An additional study reported increased breastfeeding initiation and continuation at two weeks following the introduction of the Nottingham ‘Breastfeeding Peer Support Service’^(36)^. Furthermore, another study highlighted that women receiving PS were more likely to initiate breastfeeding (*P* = 0·04)^(65)^.

Eight trials reported no statistically significant differences in breastfeeding outcomes between the IG and CG^(15,16,37,41,48,59,63,65)^. One study found similar breastfeeding initiation rates: 69·0 % (IG) *v*. 68·1 % (CG)^(16)^. Another trial reported breastfeeding initiation rates of 95 % (IG) and 96 % (CG), declining to 46 % and 42 %, respectively, by four months^(37)^. There was no difference noted in the rate of any breastfeeding at 10–14 d between the IG and CG^(15)^. In another trial, breastfeeding rates at six weeks were low in both groups, with no statistically significant difference noted (*P* = 0·07)^(41)^. Although an additional study highlighted that PS was associated with increased odds of EBF, these findings were not statistically significant^(63)^. A subsequent study found no significant differences in breastfeeding continuation at 6 weeks (*P* = 0·31) or 6 months postpartum (*P* = 0·54)^(65)^.

Overall, 50 % of the included studies (*n* 19) demonstrated that PS positively impacts breastfeeding outcomes^(32,34–36,38–40,42–47,49,60,63–65,68)^. Structured and proactive PS interventions were particularly effective in enhancing breastfeeding outcomes and maternal satisfaction with PS^(33–35,37,39,40,50,51,54,56–60,65)^. However, it is important to note that eight papers reported no significant differences in breastfeeding outcomes^(15,16,37,41,48,59,63,65)^. Several reasons may explain this, including that only 70 % of women in one study received one antenatal PS visit^(41)^, suggesting that low intervention uptake may have diluted its potential impact^(37,41)^. Many women did not receive the intended support, often due to reluctance to seek help or uncertainty about the support available^(37)^.

### Secondary outcome 2: The impact of mother-centred breastfeeding peer support on maternal emotional well-being

The impact of mother-centred breastfeeding PS on maternal emotional well-being was reported in 34 % of the included studies (*n* 13). This could suggest that support influenced mothers’ breastfeeding self-efficacy and motivation to continue breastfeeding and to progress towards achieving their personal breastfeeding goals^(33,34,43,48,51–57,59,61)^.

Mothers not only benefited from the practical and informational aspects of breastfeeding PS but also valued the emotional support^(33,34,53–57,59)^. Women appreciated feeling listened to and reassured: *‘I felt I was doing fine after talking to her’* ^(34)^. Mothers found comfort in knowing that their peer supporter was available for breastfeeding assistance, especially for new mothers who didn’t have family or friends nearby^(34)^. Mothers appreciated the ‘mother-centred’ approach, where peer supporters consistently acknowledged their feelings. They actively listened, offered encouragement and praised mothers for their breastfeeding achievements. Additionally, they helped mothers navigate the challenges of parenthood and discussed the importance of self-care, nutrition and rest ^(53)^.

Mothers appreciated the accessibility and responsiveness of peer supporters especially when professional services were not immediately available^(59)^. The use of digital platforms, such as WhatsApp, alongside home visits, enhanced accessibility by enabling more frequent and flexible contact. Several participants expressed a preference for digital communication, highlighting its potential to support programme sustainability, and reduce costs especially when face-to-face contact is not feasible^(59)^. Mothers valued how their peer supporter could relate to what they ‘were going through’ and could draw on their own experiences to provide support^(33)^. Peer supporters created a safe, non-judgmental space, with mothers describing them as *‘someone in your corner without an agenda’* ^(33)^ and even *‘like a close aunt’ ‘friend or a mother model’* ^(56)^.

The ‘Baby Cafés’ also served as a source of emotional support, where mothers could share personal challenges and receive tailored advice based on their individual needs^(54)^. One mother disclosed: *‘You know if there is anything ever…wrong, it’s almost like that friend that you can confide in…she’ll jump in with advice…she’s just there to listen and to let me offload’* ^(54)^.

Furthermore, another study highlighted how peer supporters fostered discussions on the women’s progress which instilled confidence and self-efficacy^(55)^. Women referred to their peer supporter as a *‘lifeline’*, and *‘safety net’* with one mother stating: ‘*It would have been the easiest thing in the world [to stop breastfeeding]…but when you’ve got that support there, it actually makes you stop and think twice…I can do this, they’ve done it, I can do it’* ^(55)^. Additionally, several mothers emphasised that emotional support was crucial role in helping them feel reassured and less isolated, especially during vulnerable moments in early motherhood^(57)^. One first-time mother explained how emotional distress and difficulty with latching nearly led her to stop breastfeeding, but the empathy and guidance from the peer supporter helped her persevere: *‘…they also helped with my emotions as I was a first time mum… if it hadn’t been for her I may have stopped breastfeeding…’* ^(57)^.

Several studies reported on breastfeeding self-efficacy^(43,48,51,52,55,59)^. One paper highlighted that many women, particularly those who had established careers before motherhood, felt unprepared for the unpredictability of breastfeeding^(51)^. However, once mothers received breastfeeding PS, their sense of breastfeeding self-efficacy increased^(51)^. Higher self-efficacy was observed among mothers with previous breastfeeding experience^(61)^. Additionally, a positive attitude towards breastfeeding was associated with higher initiation and continuation rates^(48)^. Overall, one-to-one breastfeeding PS significantly enhanced maternal emotional well-being by offering mother-centred, non-judgmental, empathetic care that fostered confidence, motivation and reduced feelings of isolation.

## Discussion

The findings from this review highlight that one-to-one PS have a positive impact on increasing rates of breastfeeding initiation, continuation and exclusivity, particularly in the early postpartum months^(32,34–36,38–40,42–47,49,60,63–65,68)^. Beyond breastfeeding outcomes, PS also offers critical emotional support^(33,34,53–57,59)^. Furthermore, twelve papers^(33–35,39,48,51,55–58,60,62)^ explored how PS helped mothers achieve or even extend their personal breastfeeding goals, often surpassing their initial intentions. Six papers reported on breastfeeding self-efficacy and how PS can effectively strengthen women’s confidence to initiate and sustain breastfeeding^(43,48,51,55,59,61)^. The included studies (*n* 38) employed a wide range of one-to-one PS interventions, delivered using a combination of methods such as face-to-face, telephone and digital communication, employing both structured and flexible formats.

Several intervention studies demonstrated substantial differences between PS and CG. For example, mothers receiving PS were significantly more likely to initiate and sustain EBF^(45,49)^. Regular contact with a peer supporter appeared critical for maintaining breastfeeding practices over time, as evidenced in studies reporting extended EBF rates among mothers who received ongoing PS^(39,40)^. These findings are consistent with existing evidence from Cochrane reviews^(21)^, as well as national^(5)^ and international guidance^(69,70)^ which advocate for PS as a strategy to improve breastfeeding outcomes.

Community-based support is recognised as a vital component of the Baby-Friendly Hospital Initiative^(71)^. PS within the community can enhance health literacy and help reduce health inequalities across all socio-economic groups^(72)^. Structured models such as MES and MESIGA^(38)^ along with national programmes like the Nottingham Breastfeeding Peer Support Service^(36)^ provided further evidence that well-implemented PS interventions can effectively reach and positively impact diverse populations^(36,38)^.

In light of these findings, universal access to breastfeeding PS should be a public health priority. UNICEF and WHO have called for equal access to breastfeeding support, highlighting that when mothers receive the help they need to breastfeed, the benefits extend to all. Increasing global breastfeeding rates could save more than 820 000 children’s lives annually^(73)^. This review clearly demonstrates the positive impact PS has on increasing breastfeeding rates. Only 48 % of infants under six months of age worldwide are currently EBF^(74)^. The Global Breastfeeding Collective has set ambitious targets for 2030, including 70 % EBF rates for infants under six months, 80 % breastfeeding continuation at one year and 60 % breastfeeding at two years^(75)^. Achieving these targets will require scaling up accessible and sustainable PS interventions worldwide. PS is an important tool within existing healthcare and community services to improve breastfeeding rates globally. By offering emotional encouragement, practical guidance and a sense of community, PS helps mothers navigate challenges, normalise breastfeeding practices and prolong the duration of EBF^(11)^.

However, eight papers found no statistically significant differences in breastfeeding outcomes between the IG and CG, although participants in these studies still appreciated the support they received^(15,16,37,41,48,59,63,65)^. This highlights that, even when breastfeeding outcomes are not significantly impacted, PS may provide perceived benefits that improve maternal satisfaction and confidence^(33–35,39,40,55,57,59,61,65)^. This underscores the importance of considering maternal experience as a valuable outcome in its own right, beyond traditional success metrics that evaluate the rates of breastfeeding at various time points. PS has been shown to positively influence emotional well-being, confidence and a sense of empowerment among mothers, even in the absence of measurable changes in breastfeeding rates^(76)^. Such support often helps women feel heard, respected and less isolated in their feeding journey, which can be particularly meaningful in the transition into motherhood^(77,78)^. Importantly, this reflects a shift towards a more mother-centred model of care, where success is aligned with mothers’ individual personal breastfeeding goals, rather than predetermined success metrics currently employed by public health bodies^(78,79)^. Recognising these additional benefits is key to a more holistic understanding of the support women need on their breastfeeding journey.

Overall, these findings highlight that while the measurable impact of PS on breastfeeding rates can vary across settings and modes in which support is delivered, i.e. in-person, phone calls, texts, etc., its role in empowering mothers and promoting continued breastfeeding is consistently recognised. This underscores the importance of integrating one-to-one PS into maternity and public health services to complement routine postnatal care and address gaps in emotional and informational breastfeeding support.

Society has a crucial role to play in supporting women on their breastfeeding journey through ensuring community PS programmes are accessible to them, and adequate policies are in place to support mothers. Furthermore, access to breastfeeding PS should not depend on a mother’s geographic location. All women, regardless of where they live, whether in rural or urban areas, across the island of Ireland and worldwide, should have equal access to PS. The availability of such support must be viewed as a public health priority, not a postcode lottery. Ensuring universal access to PS can help bridge disparities in breastfeeding outcomes and empower mothers to achieve their personal breastfeeding goals. For instance, a recent study from the University of Missouri highlights the challenges faced by rural mothers in accessing breastfeeding support^(80)^. Many first-time mothers in rural Missouri were initially motivated to breastfeed but discontinued within days or weeks due to limited access to breastfeeding support^(80)^. The study found that rural mothers often felt uncertain about their milk supply, lacked knowledge on latching and pumping techniques, and encountered prevailing cultural norms that favoured formula feeding over breastfeeding^(80)^. Similarly, Cueva *et al.* ^(62)^ highlighted how the frequency of interactions with peer supporters widely varied, with about 40 % of women at one site receiving only one visit from their peer supporter, whereas about half of mothers at another site received three or more visits^(62)^. These inconsistencies reinforce the urgent need for targeted support systems in rural areas as well as urban areas, including telehealth and community-based programmes, to ensure all mothers receive the guidance they need, regardless of location. In addition, breastfeeding promotion in rural areas should be strengthened through culturally sensitive health education^(81)^.

Currently in Ireland, the Health Service Executive website provides location-based guidance to help mothers access breastfeeding support services, including lactation consultants, breastfeeding clinics, maternity-based support, a live chat function in which you can access a lactation consultant online from 10.00 to 15.00 Monday to Friday and community-based support groups such as Friends of Breastfeeding, Cuidiú, and La Leche League, as well as groups facilitated by public health nurses^(82)^. These services reflect growing efforts to make breastfeeding support more accessible, particularly in community settings. Furthermore, Sláintecare^(83)^, a national healthcare reform initiative, seeks to improve healthcare accessibility and equity across Ireland. Its emphasis on delivering the right care in the right place at the right time and on shifting care into the community complements the goals of community-based breastfeeding support^(74)^.

These reforms present a valuable opportunity to integrate and strengthen breastfeeding initiatives within routine maternal care^(83)^. This review has many implications for practice and policy including the suggestion that healthcare services like the Health Service Executive consider incorporating structured, proactive, mother-centred one-to-one support programmes into routine perinatal care. It is crucial that all mothers have access to breastfeeding PS whether in the form of postnatal hospital support, home visits, phone calls, text messaging or other alternative methods of digital support.

Finally, breastfeeding PS services should be specifically tailored to meet the needs of diverse and vulnerable populations. Several studies included in this review highlighted that structured PS interventions were particularly effective among lower socio-economic groups^(38,44,46,64,68)^. For instance, data reported by Olson *et al.* ^(68)^, which was the primary source for Rozga *et al.* ^(64)^ and Clark *et al.* ^(46)^ demonstrated that targeted PS improved breastfeeding initiation and duration rates in economically disadvantaged communities, where breastfeeding rates are often lower than national averages^(46,64,68)^. Community-based breastfeeding programmes should continue to promote EBF and develop strategies to support working mothers, ensuring services are responsive to the practical challenges different groups face^(84)^. Tailoring services to the unique challenges faced by different groups is essential to achieving equitable breastfeeding outcomes and advancing public health goals.

### Implications for future research

These findings highlight several priorities for future research. While the included studies provide evidence on the impact of one-to-one PS on breastfeeding outcomes and maternal experiences, there remains limited consistency in how interventions are designed, delivered and evaluated, particularly in relation to mothers’ personal breastfeeding goals and contextual differences across settings. A more standardised evaluation of one-to-one PS interventions is needed, particularly regarding the optimal timing, frequency, duration and mode of support. Future studies should place greater emphasis on mothers’ personal breastfeeding goals and maternal satisfaction as primary outcomes, rather than relying predominantly on breastfeeding duration and exclusivity rates as the determinant of breastfeeding ‘success’. Further research should also examine the effectiveness of digital and hybrid PS models, particularly for mothers in rural or underserved communities where access to in-person breastfeeding support may be limited. There is also a notable lack of Irish-based evidence on one-to-one peer breastfeeding support, highlighting the need for future research within the Irish context.

### Strengths and limitations

The strengths of this review include its broad scope, spanning 25 years and incorporating studies from 10 countries, thereby capturing diverse global perspectives on one-to-one breastfeeding support. Guided by the framework of Arksey and O’Malley^(27)^, along with the Joanna Briggs Institute Manual for Evidence Synthesis^(31)^ and PRISMA-ScR guidelines^(26)^, this review systematically mapped the breadth of available evidence, generating key findings. By descriptively summarising a wide range of data, the review provides further insight into the positive impact of individualised breastfeeding PS in improving breastfeeding outcomes and maternal satisfaction. The inclusion of qualitative, quantitative, mixed method studies and relevant secondary data analyses aligns with the scoping review methodology, enriching the evidence base and offering a multifaceted perspective. However, this scoping review has limitations. In line with the scoping review methodology, it did not assess the quality of the included studies, which may affect the strength and reliability of the conclusions. Furthermore, the study selection process did not employ dual independent screening at the title and abstract stage, which may have increased the risk of missing relevant studies. However, multiple authors conducted full-text screening, and reference list searches were performed to mitigate this risk and reduce bias^(85)^. Restricting inclusion to English-language studies may have posed selection bias, potentially omitting relevant literature from non-English-speaking regions. This study focused on mothers’ perspectives on breastfeeding PS, which, while valuable, means the perspectives of relevant healthcare professionals and peer supporters are underrepresented. These limitations should be considered when interpreting the findings.

### Conclusion

This scoping review highlights the significant and multifaceted impact of one-to-one breastfeeding PS on maternal and infant outcomes. Notably, PS played a vital role in supporting maternal emotional well-being, fostering resilience, and enabling mothers to set, achieve, and often extend their personal breastfeeding goals. In addition, the evidence demonstrates that PS can improve the initiation, continuation and exclusivity of breastfeeding across diverse populations and settings. Structured and proactive PS interventions, delivered through various modes including face-to-face, telephone and digital support, were particularly effective in enhancing breastfeeding outcomes and maternal satisfaction.

While not all studies reported statistically significant differences in breastfeeding rates, the perceived emotional and informational benefits of PS were unanimously valued by mothers. These findings highlight that PS extends beyond measurable clinical outcomes by offering essential individualised psychosocial support that strengthens maternal confidence and reduces feelings of isolation in the postpartum period.

As highlighted previously, this review identified key knowledge gaps. In particular, there remains limited research examining attainment of mothers’ personal breastfeeding goals as a primary outcome. There is also an insufficient understanding of which specific components of one-to-one PS interventions are most effective across different populations and settings. This scoping review further highlights the absence of Irish evidence on one-to-one peer breastfeeding support and establishes a structured foundation for future context-specific research. Future research should further examine the conditions under which breastfeeding PS is most effective, including the timing, frequency, and mode of delivery, as well as the contexts in which it is provided (i.e. home, hospital, or community settings).

Recognising breastfeeding PS as a public health priority is essential. Universal, equitable access must be ensured to provide all mothers with timely, compassionate and mother-centred breastfeeding PS. Scaling up accessible and sustainable PS programmes will be critical to achieving global breastfeeding targets and improving the health and well-being of mothers and infants worldwide.

## Table 1. Long description

A table with three rows and three columns. The columns are labeled PEO, Definition, and Example. The rows are as follows: Row 1: PEO is Population, Definition is Who is my question focused on, and Example is Mothers. Row 2: PEO is Exposure, Definition is What is the issue I’m interested in, and Example is Breastfeeding goals. Row 3: PEO is Outcome, Definition is What, in relation to the issue, do I want to examine, and Example is Peer support.

Navigate back to Table 1..

## Table 2. Long description

A table with 13 rows and 2 columns. The columns are labeled ’Search terms’ and ’S1 (String 1)’ to ’S13 (String 13)’. The table lists various search terms and their corresponding strings used in a literature review. Each row provides specific search terms and their combinations using Boolean operators and truncation symbols. The search terms cover topics related to mothers, breastfeeding, goals, and peer support. The strings are used to search titles, abstracts, and keywords in multiple databases.

Navigate back to Table 2..

## Table 3. Long description

The table has two columns: Inclusion criteria and Exclusion criteria. It contains six rows of criteria each. Row 1: Mothers perspectives of one-to-one peer breastfeeding support. Healthcare professionals or breastfeeding peer supporters perspectives of one-to-one peer breastfeeding support. Row 2: Mothers who have received one-to-one peer breastfeeding support. Mothers who have only received group-based peer breastfeeding support. Row 3: Studies that focus on breastfeeding support provided by trained peer supporters or other mothers with personal breastfeeding experience. Studies focusing solely on breastfeeding support from healthcare professionals or family support. Row 4: Studies that assess how one-to-one peer support affects mothers personal breastfeeding goals, satisfaction with their breastfeeding experience and breastfeeding outcomes. Studies that do not evaluate the impact of one-to-one peer breastfeeding support on mothers personal breastfeeding goals, satisfaction or breastfeeding outcomes. Row 5: Primary research: Qualitative, quantitative and mixed method studies. Secondary research: Systematic reviews, meta-analyses, literature reviews, scoping reviews, editorials, conference abstracts and discussion papers. Relevant secondary data analysis. Irrelevant secondary data analysis. Row 6: Studies published in English. Studies published in languages other than English.

Navigate back to Table 3..

## Table 4. Long description

The table presents a structured summary of data extracted from studies on breastfeeding peer support. It includes columns for study characteristics, intervention details, and breastfeeding outcomes. Each row corresponds to a different study, with data filled in the respective columns. The table uses frequency counts and proportions to summarize quantitative data, while qualitative data is analyzed using descriptive content analysis. Key concepts identified include personal breastfeeding goals, perseverance through challenges, encouragement, emotional reassurance, and mother-centered support. These concepts are grouped into categories reflecting how one-to-one peer support influences mothers’ personal breastfeeding goals.

Navigate back to Table 4..

## Table 5. Long description

Panel A: A table listing the characteristics of included studies. The table includes columns for study author, year, country, study design, sample size, and key findings. Panel B: A table describing the interventions employed in the included studies. The table includes columns for intervention type, duration, setting, and key components. Panel C: A figure presenting a visual overview of the literature, likely including graphs or charts that summarize the data from the tables.

Navigate back to Table 5..
